# A high-efficiency differential expression method for cancer heterogeneity using large-scale single-cell RNA-sequencing data

**DOI:** 10.3389/fgene.2022.1063130

**Published:** 2022-11-29

**Authors:** Xin Yuan, Shuangge Ma, Botao Fa, Ting Wei, Yanran Ma, Yifan Wang, Wenwen Lv, Yue Zhang, Junke Zheng, Guoqiang Chen, Jing Sun, Zhangsheng Yu

**Affiliations:** ^1^ Department of Bioinformatics and Biostatistics, School of Life Sciences and Biotechnology, Shanghai Jiao Tong University, Shanghai, China; ^2^ SJTU-Yale Joint Center for Biostatistics and Data Science Organization, Shanghai Jiao Tong University, Shanghai, China; ^3^ Department of Biostatistics, Yale University, New Haven, CT, United States; ^4^ Department of Biochemistry and Molecular Biology, School of Basic Medical Sciences, Xi’an Jiaotong University, Xi’an, China; ^5^ Clinical Research Institute, Shanghai Jiao Tong University School of Medicine, Shanghai, China; ^6^ Key Laboratory of Cell Differentiation and Apoptosis of Chinese Ministry of Education, Faculty of Basic Medicine, Shanghai Jiao Tong University School of Medicine, Shanghai, China; ^7^ State Key Laboratory of Oncogene and Related Gene, Shanghai Jiao Tong University School of Medicine, Shanghai, China; ^8^ Shanghai Minimally Invasive Surgery Center, Department of General Surgery, Ruijin Hospital, Shanghai Jiao Tong University School of Medicine, Shanghai, China; ^9^ Center for Biomedical Data Science, Translational Science Institute, Shanghai Jiao Tong University School of Medicine, Shanghai, China

**Keywords:** combination test, differential analysis, colorectal cancer, PBMC68K, DE gene

## Abstract

Colorectal cancer is a highly heterogeneous disease. Tumor heterogeneity limits the efficacy of cancer treatment. Single-cell RNA-sequencing technology (scRNA-seq) is a powerful tool for studying cancer heterogeneity at cellular resolution. The sparsity, heterogeneous diversity, and fast-growing scale of scRNA-seq data pose challenges to the flexibility, accuracy, and computing efficiency of the differential expression (DE) methods. We proposed HEART (high-efficiency and robust test), a statistical combination test that can detect DE genes with various sources of differences beyond mean expression changes. To validate the performance of HEART, we compared HEART and the other six popular DE methods on various simulation datasets with different settings by two simulation data generation mechanisms. HEART had high accuracy ( $F_{1}$ score >0.75) and brilliant computational efficiency (less than 2 min) on multiple simulation datasets in various experimental settings. HEART performed well on DE genes detection for the PBMC68K dataset quantified by UMI counts and the human brain single-cell dataset quantified by read counts ( $F_{1}$ score = 0.79, 0.65). By applying HEART to the single-cell dataset of a colorectal cancer patient, we found several potential blood-based biomarkers (CTTN, S100A4, S100A6, UBA52, FAU, and VIM) associated with colorectal cancer metastasis and validated them on additional spatial transcriptomic data of other colorectal cancer patients.

## 1 Introduction

Colorectal cancer (CRC) was the world’s third most common cause of cancer mortality, with more than 850000 deaths annually (Biller and Schrag, 2021). The Colorectal cancer mortality rate was high in the setting of metastatic disease or recurrence. Predicting tumor response and selecting personalized cancer therapies based on validated biomarkers is important. Tumor heterogeneity is the major obstacle to cancer treatment (Linnekamp et al., 2015; Eide et al., 2021). Identifying differential expression genes (DE genes) associated with tumors is critical in investigating cancer heterogeneity (Soneson and Robinson, 2018; Wang et al., 2019; Kharchenko, 2021). Many differential expression analysis methods for bulk-RNA sequencing data focus on the comparison at the mean level and ignore some multi-source heterogeneities. Sequencing technologies develop rapidly, and single-cell RNA-sequencing (scRNA-seq) has become widespread in more experiments. Technological improvements in single-cell RNA sequencing drive novel biological insights and new problems in data analysis. Developments of single-cell RNA-sequencing enable researches on cancer heterogeneity at a high resolution. In contrast with bulk RNA sequencing data, the scRNA-seq data have extensive data sizes, significant fractions of observed zeros, and various gene expression patterns (Soneson and Robinson, 2018; Wang et al., 2019; Kharchenko, 2021). They are large-scale, highly sparse, variable, and complex. Emerging data features unique to scRNA-seq data require novel differential expression analysis methods to detect DE genes (Zheng et al., 2017; Ding et al., 2020).

Several DE methods for single-cell data have been proposed to fit the data characteristics in scRNA-seq data. They are two classes of methods in principle: model-based and test-based methods. Model-based DE methods model parametrically with strong assumptions of theoretical distribution of gene expression. Such as, SCDE (Kharchenko et al., 2014) assumed a mixture of Poisson (dropout) and negative binomial (amplification) distributions for the distribution of genes. DESeq2 (Love et al., 2014) tests differential expression using negative binomial generalized linear models. MAST (Finak et al., 2015) fits two-part, generalized linear models for characterizing heterogeneity in scRNA-seq data. Monocle3 (Trapnell et al., 2014; Qiu et al., 2017) uses the quasi-Poisson, or negative binomial distribution, to model gene expression counts across cells. NBID (Chen et al., 2018) calculates each gene’s independent dispersion in each group based on the negative binomial distribution. SC2P (Wu et al., 2018) supposes the gene expression with two phases and employs a zero-inflated Poisson (ZIP) distribution and a lognormal-Poisson (LNP) model to describe gene expression. Thus, the deviation between assumptive and actual distribution incurs algorithm accuracy issues. Moreover, the growth of experimental techniques requires single-cell algorithms to be scalable to handle sheer volumes of data. Large-scale, sparse single-cell data with a prevalence of zero values is challenging to model parameter convergence. Model-based DE methods have limited scalability and an evident diminution of computing performance on large-scale datasets. Statistical tests are widespread substitutions for model-based DE methods, because they have fewer assumptions and lower computing complexity than model-based methods. For example, Seurat, a popular scRNA tool, sets Wilcoxon rank-sum test as the default test to find differentially expressed genes between two groups of cells. However, tests applied for scRNA-seq data are still classical statistical tests and not grounded in biology. Classical parametric statistical tests, such as *t*-test, z-test, and F-test, have poor results due to the extreme skewness caused by the sparsity of the scRNA-seq datasets. Non-parametric tests, such as the Wilcoxon rank-sum test, adapt for the sparsity of scRNA-seq data. But, they have awful accuracy because of the high heterogeneity and complexity of scRNA-seq data. The probabilities of Type 1 errors of the non-parametric tests vary systematically with the increasing heterogeneous variances and remain relatively constant even if the sample size increases (Zimmerman, 2000). Furthermore, non-parametric tests focus more on locations than the distribution shape, so they cannot sensitively capture various biological differences in scRNA-seq data. Each of these two types of methods has its advantages and limitations. Existing DE methods, whether model-based or test-based, have difficulty balancing accuracy and computational efficiency simultaneously in large-scale single-cell data.

In this study, we present HEART, a scalable combination test for DE analysis of single-cell data. Underlying this test framework, HEART can sensitively detect biological differences in gene expression beyond mean expression shift. We illustrate the benefits of HEART *via* comparing the performances of HEART and the other six DE methods (DESeq2 (Love et al., 2014), MAST (Finak et al., 2015), Monocle3 (Trapnell et al., 2014; Qiu et al., 2017), NBID (Chen et al., 2018), SC2P (Wu et al., 2018), Seurat) on vast simulation experiments based on two simulation generation mechanisms. HEART performs well in accuracy, scalability, statistical robustness and computational efficiency. We demonstrated that HEART performs robustly on two real single-cell datasets underlying different quantification schemes. Furthermore, we applied HEART to a single-cell dataset of a colorectal cancer patient and identified several potentially metastasis-related biomarkers, CTTN, S100A4, S100A6, etc.

## 2 Results

### 2.1 HEART overview

**FIGURE 1:**
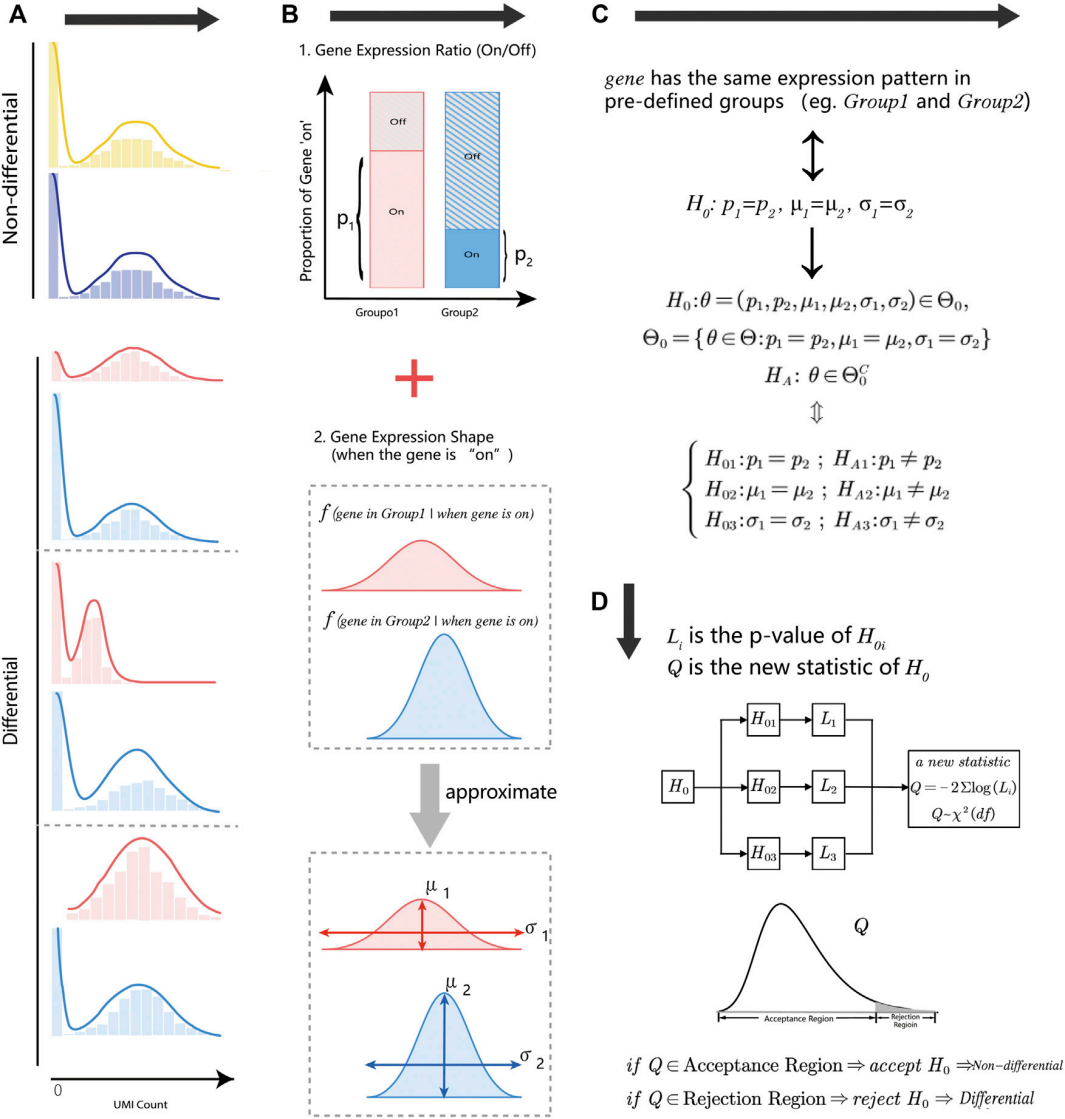
An overview of the HEART. **(A)** Diagram of non-differential and differential gene expression patterns. The non-differential genes have the same statistical distribution in different groups. Differential gene expression patterns have several modes with different characteristics. **(B)** The gene expression distribution decomposes into two parts: the gene expression state (“On/Off”) and the gene expression shape when the gene is “On.” Two parameters could approximate the distribution shape of the gene “On” part: the location parameter μ and the scale parameter σ . **(C)** HEART’s combination statistical test structure. Combination test flow chart.

Droplet-based single-cell RNA-sequencing methods measure gene expression on tens or hundreds of thousands of cells at the single-cell level. Gene expression measurements in droplet technology are often in the form of low counts with a large fraction of zero values, and difficult to estimate the exact statistical distribution. We decomposed the gene expression distribution into two parts (Figure 1B): the status of genes (“on/off”) and the distribution shape of gene “on” parts (non-zero part). These two parts were closely associated with cell type, cell condition, or other biologic-driven factors. For the first part, the gene expression state ratio was defined as the times of the gene with the positive count in a group of cells. For the gene “On” part, we described the distribution shape by location parameter ( μ ) and scale parameter ( σ ) of the “On” parts (Figure 1B). Therefore, the whole gene expression pattern can be approximated by three parameters: the zero proportion of gene expression ( p ), the mean of the “On” parts ( μ ), and the variance of the “On” parts ( σ ).We assumed that non-DE genes have the same expression distribution shape in pre-defined groups (Figure 1A). We tested three parameters ( $H_{0}:p_{j1}=p_{j2},\;\mu_{j1}=\mu_{j2},\;\sigma_{j1}^{2}=\sigma_{j2}^{2}$ ) to identify whether a given gene is a DE gene (Figure 1C). Due to low counts, sparsity, and complexity of gene expression, it is challenging to estimate the exact distribution of every gene and construct a suitable statistic for the hypothesis $H_{0}$ when the theoretical distribution of genes is unknown. Instead of generating the test statistic based on the assumed distribution, we tested the complex null hypothesis $H_{0}$ using Fisher’s (Zappia et al., 2017) theory of combination test.

$$H_{0}:\;\theta =\left(p_{j1},\;p_{j2},\;\mu_{j2},\mu_{j2}.\sigma_{j1},\sigma_{j2}\right)\in \Theta_{0},\;\Theta_{0}=\left\{\theta \in \Theta :p_{j1}=p_{j2},\mu_{j1}=\mu_{j2},\;\;\sigma_{j1}=\sigma_{j2}\right\}$$

$$H_{A}:\;\theta \in \Theta_{0}^{C}$$

$$\left\{ \begin{array}{l} H_{01}:p_{j1}=p_{j2};\;H_{A1}:p_{j1}\neq p_{j2}\\ H_{02}:\mu_{j1}=\mu_{j2};\;H_{A2}:\mu_{j1}\neq \mu_{j2}\\ H_{03}:\sigma_{j1}^{2}=\sigma_{j2}^{2};\;H_{A3}:\sigma_{j1}^{2}\neq \sigma_{j2}^{2} \end{array}\right.$$

We split the complex null hypothesis $H_{0}$ into three simple null hypotheses $H_{0i}$ and got a new statistic $Q=-2\sum \log\left(L_{i}\right)$ by combining three individual *p*-values $L_{i}$ . Each *p*-value $L_{i}$ was obtained by testing the simple null hypothesis $H_{0i}$ . The chi-square distribution was used to approximate the *p*-value of Q . Underlying this test framework, we easily captured various differences in gene expression and constructed a test for gene expression patterns without many assumptions. Moreover, we only calculated three simple observed test statistics and got the new statistic Q by combining three individual *p*-values $L_{i}$ . We could quickly identify differential expression (DE) genes in millions-scale scRNA data. The computation cost is almost negligible. If the new statistic Q is larger than the critical value, we reject the null hypothesis and identify the gene as a DE gene. We examined one gene at a time and implemented FDR correction for *p*-values of all genes.

### 2.2 HEART validation

HEART proposed a combination test to catch various sources of differences in gene expression patterns between two pre-defined groups. To validate the performance of HEART, we used two simulation data generation mechanisms to compare HEART and other six popular DE methods, including five model-based DE methods (DESeq2, MAST, Monocle3, NBID, and SC2P) and a default test in Seurat (Seurat-W). Simulation details were provided in the “Methods”. Briefly, the artificial simulation tool, Splatter package (Zappia et al., 2017), generated datasets in simulation1. Simulation2 datasets used a semi-simulation mechanism based on actual scRNA-seq data (PBMC68K)to create simulation datasets. In both simulations, we varied the number of samples and DE strength for DE genes. We evaluated the ability to identify DE genes, FDR control under the null hypothesis, and computational efficiency under various alternatives by a series of indexes: $F_{1}$ score, TPR, precision, computational time, etc.

**FIGURE 2:**
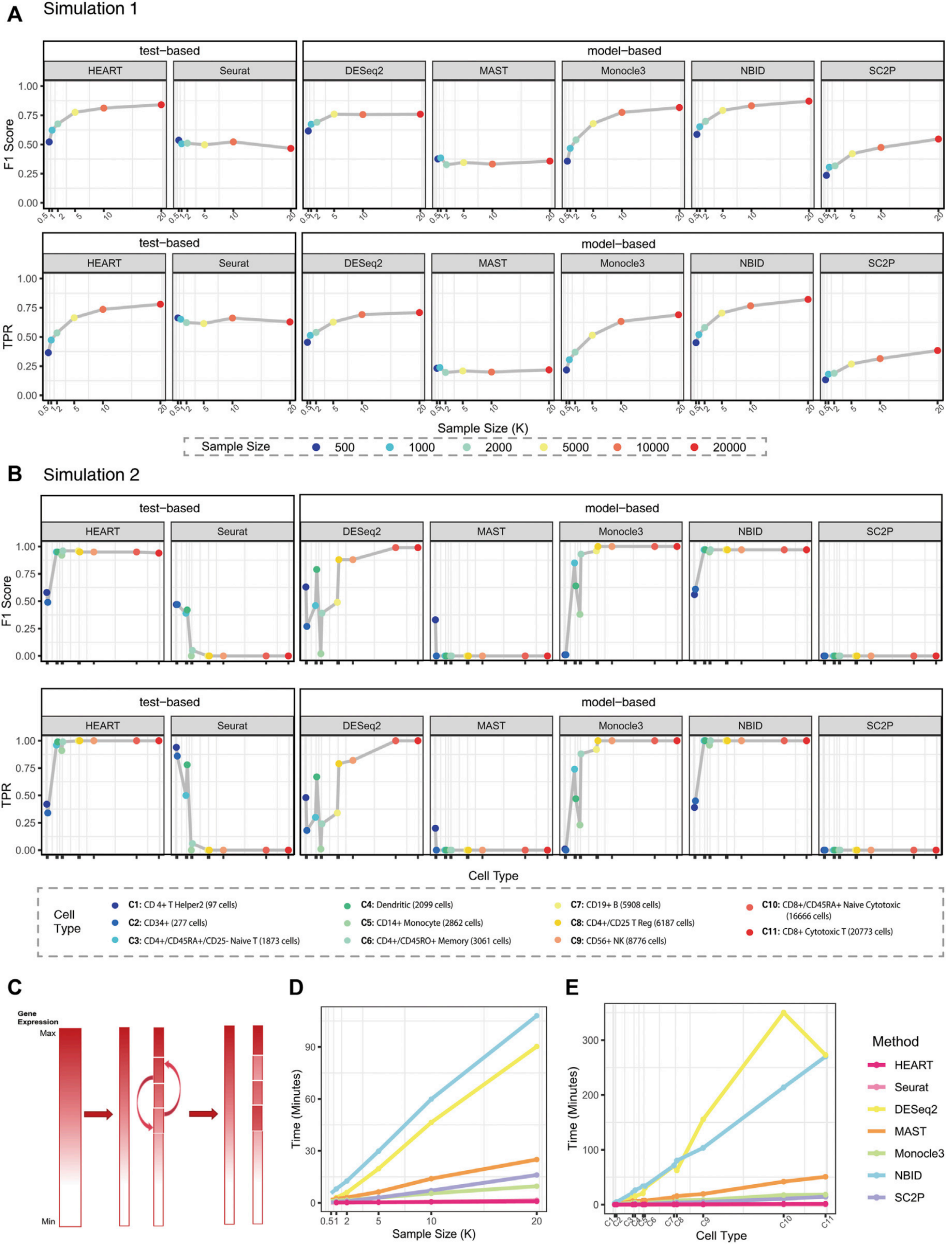
Simulation results. **(A)** $F_{1}$ scores and TPRs of all methods on simulation datasets in Simulation 1 (de.factor = 0.5). Plots show $F_{1}$ scores (y-axis) and TPRs (y-axis) for different sample sizes (x-axis) for different methods. Colorful points correspond to varied sample sizes. **(B)** $F_{1}$ scores and TPRs of all methods on simulation datasets in Simulation 2 (FC = 2.5). Plots show $F_{1}$ scores (y-axis) and TPRs (y-axis) for different source data (x-axis) for different methods. Colorful points correspond to different source datasets with different cells. **(C)** Semi-simulation data generation mechanism in Simulation 2. **(D)** and **(E)** Computational time of different methods for analyzing data with different sample sizes in Simulation1 and Simulation 2, respectively. The X-axis in **(E)** corresponds to the legend of **(B)**.

In simulation 1, we evaluated the performances of each method on simulation datasets with the same simulation settings. HEART, Monocle3, and NBID perform better than other methods (Figure 2A; Supplementary Figure S2). They had higher $F_{1}$ scores than other methods and achieved a good balance between TPR and precision. Seurat had low precisions, because it was apt to identify the gene with mild signals. DESeq2 maintained high accuracy on medium-scale data (under 10000 cells), but it shows FDR inflation on the large-scale datasets (Supplementary Figure S2). Regarding running time, HEART and Seurat had incomparable advantages (Figure 2D, under 2 min on the datasets of 20000 cells). Although NBID and DESeq2 had good accuracy, they required a lot of running time (Figure 2D, more than 1 h on the datasets of 20000 cells with 11000 genes).

In Simulation2, we generated semi-simulation data from real scRNA-seq datasets instead of simulation datasets from artificial protocols (Figure 2B; Supplementary Figure S3) (Chen et al., 2018). We chose each cell subtype with various sample sizes from PBMC68K (Zheng et al., 2017) as source data to test the stability and scalability of each DE method. HEART, NBID, and Monocle3 have higher $F_{1}$ scores in different simulation datasets than other methods. When the sample size was adequate, HEART had good and stable performances, regardless of the statistical characteristics of the datasets. Seurat performed unstably on different datasets. DESeq2, MAST, and SC2P cannot detect DE genes in most scenarios. Importantly, HEART was much more computationally efficient than the other methods (Figure 2E). For the 20000-cells scale datasets, HEART completed computation in about 1–2 min, but NBID and DESeq2 needed 5–7 h for the same scale datasets. HEART was applicable to data with the sample size exceeding around millions of cells in theory. We generated null simulations without swapping genes to test the bias in *p*-value estimation for each method (Supplementary Figure S4). HEART controlled the type 1 error well.

**FIGURE 3:**
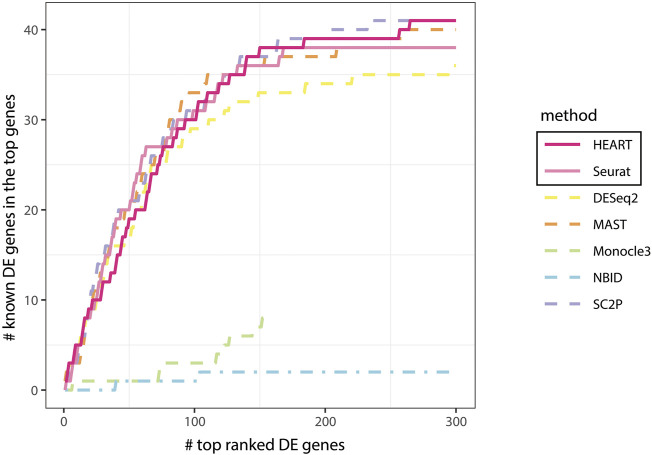
Comparing all methods: known DE genes among the top ranked DE genes in human brain cells for astrocytes and oligodendrocytes cells.

Generally, HEART was an accurate, practical and scalable method for DE gene detection. In all semi-simulation scenarios, HEART and NBID performed better than other methods and had relatively stable performances on datasets with various characteristics. Other methods had poor performances on some semi-simulation datasets. As the sample size increases, the performances of HEART, NBID, and Monocle3 become better. However, HEART identified DE genes in the simulation scenarios with weak DE strength of differences, which means HEART was more sensitive than other competing DE methods (Supplementary Figure S3; Figure 3). The performance of NBID was slightly better than HEART in some scenarios, but it took a lot of time to run. (Simulation1 of 20000 cells: NBID: $F_{1}$ score = 0.871 running time = 6482 s; HEART: $F_{1}$ score = 0.84, running time = 52 s. Simulation2 of CD8^+^ cytotoxic T cells: NBID: $F_{1}$ score = 0.97, running time = 16205 s; HEART: $F_{1}$ score = 0.94, running time = 94 s)

### 2.3 HEART is accurate and robust on read and unique molecular identifier counts data

Read count and unique molecular identifier (UMI) count are two main quantification schemes in single-cell RNA-sequencing technologies and have different statistical characterizations. Some literature (Zilionis et al., 2017; Chen et al., 2018; Kashima et al., 2020; Sarkar and Stephens, 2021) suggested that read count data have higher count levels, more sparsity and more variability than UMI counts data. To assess the accuracy and robustness of HEART on different quantification mechanisms, we applied HEART and other six DE methods (Seurat, DESeq2, MAST, Monocle3, NBID, and SC2P) on two real single-cell datasets from quantification schemes. A human brain dataset (Darmanis et al., 2015) (GSE67835) based on read count quantification schemes and a dataset of peripheral blood mononuclear cells (PBMC68K (Zheng et al., 2017)) quantified by UMI counts.

#### 2.3.1 Performances on human brain data

**FIGURE 4:**
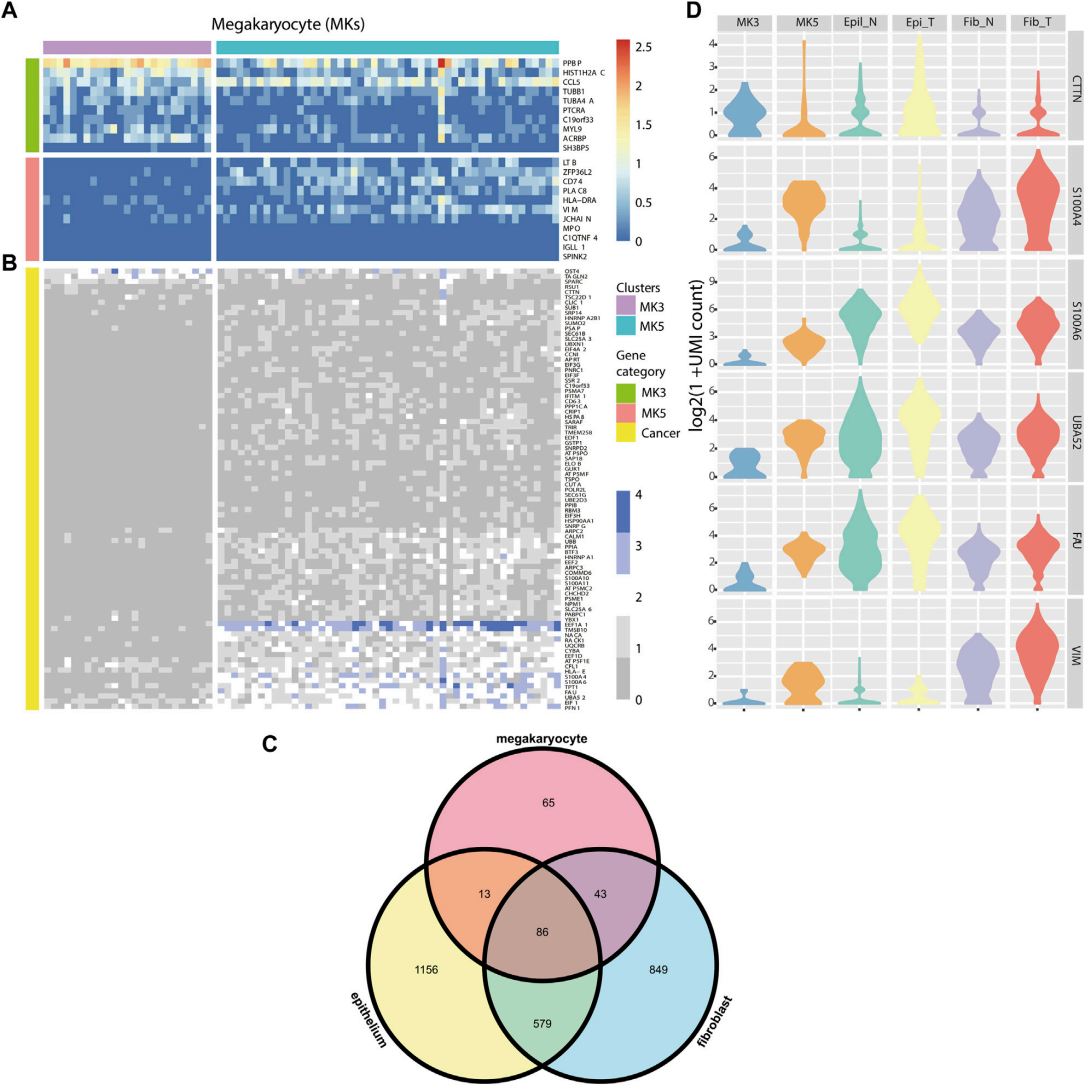
**(A)** Heatmap of marker genes for MK3 and MK5 clusters. **(B)** Heatmap of some DE genes detected by HEART. **(C)** Venn diagram of DE genes from 3 cell types of tumor and normal cells. **(D)** Violin plots showing some DE genes’ expression patterns in MK3, MK5 cluster, Epithelial cluster, and fibroblast cluster in tumor and normal tissues, respectively.

Human brain data (GSE67835) (Darmanis et al., 2015) was a single-cell dataset quantified by read count. It sequenced 466 cells from human cortical tissue containing six sub-cell types. In this human brain data, we used all seven DE methods to identify DE genes on two groups of cells (astrocytes: 62 cells, oligodendrocytes: 38 cells) with 10483 genes. The number of DE genes of different DE methods varied greatly (Table 1). At an FDR of 5%, HEART identifies 973 DE genes. For Standard 1, we obtained a list of 41 DE genes (Standard 1) between these two sub-celltypes by comparing purified cell types *via* bulk RNA-seq (Zhang et al., 2014; Darmanis et al., 2015). DE genes identified by HEART cover all 41 DE genes in Standard 1. NBID and SC2P also identified 41 DE genes in Standard 1. Still, they identified too many genes as DE genes (NBID: 6116 DE genes, SC2P: 2220 DE genes) and had low specificities (NBID: specificity = 0.42, SC2P: specificity = 0.79), suggesting potentially false signals. Underlying Standard 2 (top 500 genes) and Standard 3 (top 1,000 genes), HEART had the highest $F_{1}$ scores and relatively high TPRs and specificities compared to other DE methods (Table.1). Moreover, we compared the ability of the 41 DE genes detected in the literature from the top ranked DE gene reported by each method (Figure 4). Figure 4 showed that HEART, MAST, and SC2P have higher sensitivity and reliability in capturing true DE signals than the other four DE methods.

**TABLE 1:** The time consumption, number of DE genes, TPR, specificity, and $F_{1}$ score of each method under three different standards (Human brain data).

Method	Time (s)	#(DE genes)	Standard 1			Standard 2			Standard 3		
			TPR	Specificity	$F_{1}$ score	TPR	Specificity	$F_{1}$ score	TPR	Specificity	$F_{1}$ score
HEART	9.74	973	1.00	0.91	0.08	0.96	0.95	0.65	0.96	0.95	0.65
Seurat	10.79	2,943	0.93	0.72	0.03	0.83	0.75	0.24	0.83	0.75	0.24
DESeq2	75.36	5,814	1.00	0.45	0.01	0.99	0.47	0.16	0.99	0.47	0.16
MAST	82.35	2,155	0.98	0.80	0.04	0.99	0.83	0.37	0.99	0.83	0.37
Monocle3	60.73	154	0.20	0.99	0.08	0.29	1.00	0.45	0.29	1.00	0.45
NBID	263.01	6,116	0.80	0.42	0.01	0.99	0.44	0.15	0.99	0.44	0.15
SC2P	27.28	2,220	1.00	0.79	0.04	0.99	0.83	0.36	0.99	0.83	0.36

In this human brain single-cell dataset quantified by read counts, HEART performs best among seven DE methods. Underlying different standards, HEART always had excellent accuracy for DE gene detection. DESeq2 and NBID had high TPRs, but they maybe detect false DE genes because they identified overabundant genes as DE genes.

#### 2.3.2 Performances on PBMC68K

PBMC68K (Wang et al., 2019) was a single-cell UMI count dataset of peripheral blood mononuclear cells (PBMCs) generated by 10X Genomics. T cells were the most abundant cell type in PBMCs and play an essential role in the immune response and immune regulation. Naïve T cells and memory T cells had significant differences in functions and features, but they had a large degree of similarity in their overall gene expression (Supplementary Material S1; Supplementary Figure S1). The researches on gene expression patterns of the two types of T cells were still inadequate (Liu et al., 2001; Weng et al., 2012). We used all seven DE methods (HEART, Seurat, DESeq2, MAST, Monocle3, NBID, and SC2P) to detect DE genes between CD4^+^ Naive T cells (1873 cells) and CD4^+^ memory T cells (3,061 cells) from the PBMC68K (Zheng et al., 2017) dataset with 12406 genes. The number of DE genes identified by each method is very different. HEART, Seurat, DESeq2, MAST, Monocle3, NBID and SC2P selected 692, 676, 459, 36, 431, 1,214, 121 genes, respectively (Table.2). For Standard 1, 37 known DE genes from the literature were obtained from various microarray experiments of T cells from both humans and mice (Liu et al., 2001; Weng et al., 2012). None of the DE methods in our research fully identified these 37 true DE genes. HEART, Seurat, DESeq2, Monocle3, MAST, NBID, and SC2P captured 12, 16, 0, 28, 9, 4, 20 DE genes (Table.2), respectively. NBID detected most DE genes of the standart1, but it identified the most gene (1,214 genes) as DE gene. HEART and Monocle had relatively higher TPR, specificity, and $F_{1}$ scores than other methods (Table.2). Note that some genes with very low expression, such as gene FAS, TNF, (average UMI count in two groups: 0.017, 0.028), were only detected by HEART and the NBID. Underlying Standard 2 and Standard 3, HEART had higher $F_{1}$ score (0.77 and 0.79) than other test-based DE methods and most model-based DE methods (Table.2). HEART had high TPRs while ensuring high specificity. Moreover, on the datasets of thousands of cells, HEART only needed 40 s to run, while DESeq2 and NBID took an hour. In this application of real-data DE analysis, HEART had good accuracies assessed by different standards and spends a short running time. Especially compared with the test-based method, Seurat, HEART performed better. Compared with model-based DE methods, HEART had higher $F_{1}$ scores than most model-based DE methods and ran faster than all model-based DE methods.

**TABLE 2:** The time consumption, number of DE genes, sensitivity, specificity, and $F_{1}$ score of each method under three different standards (PBMC68K).

Method	Time (s)	#(DE genes)	Standard 1			Standard 2			Standard 3		
			TPR	Specificity	$F_{1}$ score	TPR	Specificity	$F_{1}$ score	TPR	Specificity	$F_{1}$ score
HEART	40	692	0.54	0.95	0.05	0.92	0.98	0.77	0.67	1.00	0.79
Seurat	7	676	0.24	0.95	0.03	0.71	0.97	0.60	0.52	0.99	0.62
DESeq2	3,345	459	0.32	0.96	0.05	0.83	1.00	0.87	0.46	1.00	0.63
MAST	753	36	0.00	1.00	0.00	0.00	1.00	0.00	0.00	1.00	0.00
Monocle3	290	431	0.43	0.97	0.07	0.81	1.00	0.87	0.43	1.00	0.60
NBID	3,905	1,214	0.76	0.90	0.04	0.97	0.94	0.56	0.85	0.97	0.77
SC2P	223	121	0.11	0.99	0.05	0.23	1.00	0.37	0.12	1.00	0.22

### 2.4 HEART identifies metastatic colorectal cancer biomarkers

Colorectal cancer (CRC) is the most commonly diagnosed cancers in the world.20% of individuals with newly diagnosed colorectal cancer have metastatic disease upon presentation, and another 25% of those who initially have localized illness will eventually acquire metastases (Biller and Schrag, 2021). Distant metastasis was the main cause of death in patients with colorectal cancer, but the exact metastasis mechanism was still unknown. (Zhang et al., 2014). ScRNA-seq technology provided a new opportunity to investigate the association between genes and the mechanism of tumor initiation, progression, and metastasis (Lawson et al., 2018). Therefore, we applied HEART in a single-cell dataset (containing three sub-datasets: PBMC, normal tissue, and tumor tissue) of a stage III colorectal cancer patient. We used HEART to identify DE genes between tumor and normal fibroblasts and between tumor and normal epitheliums. Furthermore, we found two subpopulations of megakaryocytes (MKs) (Wang et al., 2021) in the PBMCs and utilized HEART to detect 207 DE genes on the2 MK subtype clusters to characterize functional differences and underlying molecular mechanisms. Highly expressed genes in the cluster MK3 (Satija et al., 2015; Stuart et al., 2019; Fa et al., 2021; Wang et al., 2021), such as CCL5, TUBB1, MYL9, HIST1H2AC, etc. (Figure 4A), were associated with early platelet production. Another subpopulation, MK5, with high CD74 and PLAC8 might be a less mature MK population. Moreover, we observed that many DE genes between MK3 and MK5 cells overlap with DE genes between tumor and normal epitheliums and DE genes between tumor and normal fibroblasts (Figure 4B **,** Figure 4C). They had similar expression patterns in the MK5 cells, tumor epitheliums, and fibroblasts (Figure 4D) and were related to colorectal cancer progression or metastasis. The Violin plot showed similar distribution shapes of CTTN in MK5 cells and epithelial tumor cells. The gene CTTN has been reported overexpressed in various cancers, including colorectal cancer, and had the function of promoting tumor cell migration (Luo et al., 2006; Jing et al., 2016; Zhang et al., 2017). Furthermore, S100A4 (Helfman et al., 2005; Nader et al., 2020), S100A6 (Komatsu et al., 2000), UBA52 (Zhou et al., 2019), FAU (Pickard et al., 2011), and VIM (Luque-Garcia et al., 2010; Xu et al., 2017), etc. Also had similar expression patterns between MK5 cells and tumor epitheliums and fibroblasts. S100A4 and S100A6 play an important role in tumor metastases, including colorectal tumor metastasis (Komatsu et al., 2000). Recent studies have proved that the bloodstream plays a crucial role in tumor metastasis and tumor immune escape (Lawson et al., 2018). The cooperation of hematopoiesis, megakaryocytes, and platelet production aided CTCs in escaping the immune system and disseminating within the bloodstream to establish distant organ metastasis. We also validated the expression pattern of these genes in the spatial transcriptome data of two other stage IV colorectal cancer patients (Supplementary Figure S4), which showed spatial patterns of high expression in cancer cells.

Consequently, we supposed that a series of genes, CTTN, S100A4, S100A6, etc., were potential colorectal cancer metastasis biomarkers. The MK5 subpopulation with highly-expressed above potential biomarkers might be a cluster related to colorectal cancer metastasis and have a circulating tumor cell (CTC). The exact mechanism between MK5 and colorectal tumor metastasis warranted further investigation.

## 3 Discussion

Differential expression analysis was a crucial topic in cancer heterogeneity analysis. The new characteristics of scRNA-seq data put forward new challenges for the DE method. Model-based methods methods’ performances are unstable due to strong assumptions and lacked scalability facing the explosive growing scale of single-cell data. Test-based methods were more scalable than model-based methods. However, the accuracy existing in these test-based methods was relatively too low in identifying DE genes due to the sparsity, variability, and complexity of scRNA-seq data. HEART proposed a bio-driven combination test framework that captures comprehensive differences by integrating differential information about gene expression ratio, gene expression level, and variability. Unlike most competitors assuming theoretical statistical distribution (some are complex mixture distributions) for gene expression, HEART used a combination framework of simple statistical tests to test the two parts of the gene expression. We compared HEART and the other six DE methods on various simulation datasets with different sample sizes and DE strength of DE genes. HEART achieved an excellent trade-off between accuracy and computational efficiency. It had higher $F_{1}$ scores than all classical test method and most model-based methods and can be apt to expand to ultra-large-scale of datasets. Moreover, HEART had robust performances facing datasets with different statistical characteristics, while DESeq2 and Monocle3 had unstable performances on diverse datasets. Although NBID acted better than HEART in some scenarios, its computational cost on large-scale data sets may not be worth the increased accuracy it provided (A dataset with 20000 cells and 10000 genes: NBID: $F_{1}$ scores = 0.871, running time = 6482 s; HEART: $F_{1}$ scores = 0.84, running time = 52 s). To demonstrate the accuracy, robustness, and generality of HERAT, we compared HEART and the other six DE methods on two single-cell datasets from different quantitative mechanisms. HEART had high accuracy and low specificity on two various quantification forms data. We applied HEART and other six methods to identify DE genes between CD4^+^ Naive T cells and CD4^+^ memory T cells from the PBMC 68k dataset quantified by UMI counts. HEART had less computational time and higher TPRs and $F_{1}$ scores than other methods under different standards. Moreover, HEART had a good ability to capture the DE gene with low expression counts level, which is easily omitted in most DE analysis methods. HEART identified gene FAS and TNF, verified DE genes in literature, with lower gene expression ratios and expression counts in this PBMC68K dataset. On human brain single-cell datasets quantified by read count, HEART had the highest accuracy and controls false-positive rates well. It achieved a good balance between sensitivity and specificity. In addition, applying HEART on two subpopulations of megakaryocytes, we found several potential cancer biomarkers (CTTN, S100A4, S100A6, UBA52, FAU, and VIM, etc.) associated with colorectal cancer progression and metastasis in literature. HEART also detected these DE genes between normal and tumor epitheliums and fibroblasts. We observed the expression pattern of these genes showed spatial patterns of high expression in cancer cells in the spatial transcriptome data of two other stage IV colorectal cancer patients. Megakaryocytes are the source of platelets. Whereas the contribution of platelets to cancer procession and metastasis has been extensively characterized (Cho et al., 2012), the interaction of tumor cells with platelets and megakaryocytes during the metastatic cascade was less well-defined. Currently, the role of megakaryocytes during metastasis was starting to be appreciated. Some studies have demonstrated that increasing number of megakaryocytes in patients with cancer metastases (Huang et al., 2019; Lucotti and Muschel, 2020). In recent years, studies about platelet and megakaryocytes transcriptome at the single-cell level indicated that megakaryocytes and platelets are much more diverse than before. They fulfilled their distinct functions by utilizing heterogeneous subpopulations (Kharchenko, 2021; Liu et al., 2021). Keeping with these studies, we found that an MK subpopulation correlated with colorectal cancer metastasis. Furthermore, the proven colorectal cancer biomarkers had similar gene expression patterns in MK5 subpopulation cells and tumor epitheliums. The correlation between the MK5 subpopulation and colorectal cancer metastasis may be closer than previous studies. Of course, the comprehensive link and the underlying molecular basis between MKs, platelets, and tumor cells need more experiments and research to clarify. HEART has two main limitations: first, it is sensitive to sample size similar to other DE methods and performs poorly on small datasets ( $n_{1}+n_{2}<60$ ). Second, HEART is only designed for comparisons between two groups, and expansion to comparisons between multiple groups requires more research.

In summary, HEART is a competitive DE method for scRNA-seq data, which maintains high accuracy, unrivaled computational efficiency, and strong robustness across diverse scRNA-seq datasets.

## 4 Materials and methods

### 4.1 Datasets

We used three actual scRNA-seq datasets in applications. The PBMC68K is available from https://support.10xgenomics.com/single-cellgene-expression/datasets. The human brain dataset can be obtained by R package SC2P or the GEO database repository under accession code GSE67835. The scRNA-seq data of one stage III colorectal cancer patient have been deposited in the OMIX, China National Center for Bioinformation / Beijing Institute of Genomics, Chinese Academy of Sciences (https://ngdc.cncb.ac.cn/omix: accession no. OMIX002120). The spatial transcriptomic data of two colorectal cancer patients are available from (http://www.cancerdiversity.asia/scCRLM).

### 4.2 Simulation settings

We used two simulation data generation mechanisms to generate scenarios with different settings. Each design had 20 replications. The popular artificial protocol, Splatter (Zappia et al., 2017), generated simulation datasets in simulation 1. Each scenario contained 10000 genes (1000 DE genes and 10000 non-DE genes) and two underlying subpopulations. We varied the number of samples (1,000, 2000, 5,000, 10000, 20000) and DE strength for DE genes (de.factor = 0.3, 0.5). De.factor is the differential expression factor produced from a log-normal distribution. A high de.factor can result in the strong DE strength of DE genes between groups (More details of parameters in Supplementary Material S1).

Simulation 2 adopted a semi-simulation mechanism based on actual scRNA datasets to recover the multimodality and biological characteristic complexity of actual scRNA-seq data (Figure 2C, Supplementary Material S1) (Chen et al., 2018). First, we randomly divided the real scRNA-seq dataset into two parts regarded as two groups of cells. The second step was to create differentially expressed genes. We ranked the mean counts of all genes of the second group of cells and chose 200 genes, starting with the one having a mean count just above $s_{1}$ . We selected another 200 genes beginning with the mean count just above $s_{2}=FC\times s_{1}$ . Then, we swapped the gene expression of these two equal numbers sets of selected genes in the second group of cells and got a simulation dataset with 2 cell groups with a known DE genes list. The parameter FC controlled the DE strength of DE genes between groups. We considered three DE strengths of DE genes: weak (FC = 1.5), moderate (FC = 2), and strong (FC = 2.5). In simulation 2, we chose PBMC68K (Zheng et al., 2017) as source data. PBMC68K consisted of transcription profiles of −68000 peripheral blood mononuclear cells and had 11 different cell subtypes with sample sizes ranging from −90 to −20000 (more details in Supplementary Material S1). We generated three simulation scenarios for each subtype of cells with three different levels (weak, moderate, and strong) of difference to test the sensitivity of detecting the DE genes.

### 4.3 DE genes list

All DE gene lists in simulation datasets were artificially set. We calculated all method performance indices according to known DE gene lists. Due to the unattainability of the whole accurate DE genes list of different cell groups in real single-cell data, we used different standards to set three potential DE gene lists and calculated all method performance indices.

Standard 1. Known DE genes from the literature.

Standard 2. The top 500 genes are ranked by the chosen number of times by all methods.

Standard 3. The top 1,000 genes are ranked by the chosen number of times by all methods.

For Standard 1, we collected dozens of known DE genes from various experiments based on bulk RNA-seq in the literature (Liu et al., 2001; Weng et al., 2012; Zhang et al., 2014; Darmanis et al., 2015). They were partial genes of the whole true DE genes between different cell clusters. For Standard 2 and 3, we ranked all genes’ chosen number of times by all methods and set the top 500 and 1,000 genes as potential DE genes between different cell clusters.

### 4.4 Index

On the basis of the DE gene list in 2.3, we calculated a series of indices: $F_{1}$ scores, true positive rate (TPR, recall), false discovery rate (FDR), and time consumption to assess the performance of all methods. All indices were presented as the average value of 20 replications.

### 4.5 Method details

For this $H_{01}$ , we compared the positive expression ratio of the gene j in the two groups of cells. The total numbers of two groups of cells are $n_{1}$ , $n_{2}$ , respectively. And the numbers of positive expressions of the gene j in the two groups of cells are $m_{j1}=\sum I\left(y_{j1i}\neq 0\right)$ , $m_{j2}=\sum I\left(y_{j2i}\neq 0\right)$ , respectively. $y_{jgi}$ denotes the UMI count of the gene j of cell i in the group g=1,2 . $p_{jg}$ is the gene j ’s positive expression proportion in the group g . ${\hat{p}}_{jg}$ is the estimator of $p_{jg}$ . Hence, the positive expression ratios of the gene j in *group 1* and *group 2* are ${\hat{p}}_{j1}=\frac{m_{j1}}{n_{1}}$ , ${\hat{p}}_{j2}=\frac{m_{j2}}{n_{2}}$ , respectively.

$$H_{01}:p_{j1}=p_{j2};H_{A1}:p_{j1}\neq p_{j2}$$

$$z=\frac{{\hat{p}}_{j1}-{\hat{p}}_{j2}}{\sqrt{{\hat{p}}_{*}\left(1-{\hat{p}}_{*}\right)\left(\frac{1}{n_{1}}+\frac{1}{n_{2}}\right)}}∼N\left(0,1\right)$$

$${where,\hat{p}}_{*}=\frac{m_{j1}+m_{j2}}{n_{1}+n_{2}}$$

$$L_{1}=2P\left(Z>\left|z\right|\;|\;H_{01}\;is\;true\right)$$

In terms of hypotheses $H_{02}$ and $H_{03}$ , only the “**On**” state of each gene is involved in calculations. For hypothesis $H_{02}$ , we used the Student’s t-test to determine whether the two groups differ significantly on the central location of gene j ’s expression of the “**On**” state.

$$H_{02}:\mu_{j1}=\mu_{j2};H_{A2}:\mu_{j1}\neq \mu_{j2}$$

$$t=\frac{{\bar{x}}_{j1}-{\bar{x}}_{j2}}{\sqrt{\frac{s_{j1}^{2}}{m_{j1}}+\frac{s_{j2}^{2}}{m_{j2}}}}∼t\left(df_{t}\right)$$

$$L_{2}=2P\left(T>|t|\;|\;H_{02}\;is\;true\right)$$

$\mu_{jg}$ is the mean of the gene j in the group g on the positive part (“on” state). ${\bar{x}}_{jg}=\frac{\sum x_{jgi}}{m_{jg}}$ is the estimator of the $\mu_{jg}$ . $s_{jg}$ is the estimator of the $\sigma_{jg}^{2}$ , which is the variance of the gene j in the group $g^{\prime }s$ ‘on’ part.Where, $x_{jg}=\left\{y_{jgi},which\;y_{jgi}>0\right\}$

For this $H_{03}$ , we used the Brown–Forsythe test to test the equality of scattering of gene j ’s positive expression.

$$H_{03}:\;\sigma_{j1}^{2}=\sigma_{j2}^{2};\;H_{A3}:\;\sigma_{j1}^{2}\neq \sigma_{j2}^{2}$$

$$W=\frac{\sum_{g=1}^{G}\left(m_{jg}-1\right)}{G-1}\frac{\sum_{g=1}^{G}m_{jg}{\left({\bar{z}}_{jg}-{\bar{z}}_{j}\right)}^{2}}{\sum_{g=1}^{G}\sum_{i=1}^{m_{jg}}{\left(z_{jgi}-{\bar{z}}_{jg}\right)}^{2}}∼F\left(G-1,\;\overset{G}{\underset{g=1}{\sum }}\left(m_{jg}-1\right)\right),\;z_{jgi}=\left|x_{jgi}-{\tilde{x}}_{jg}\right|$$

$$L_{3}=P\left(F\left(1,m_{j1}+m_{j2}-2\right)>W\;|H_{03}\;is\;true\right)$$

Where, ${\tilde{x}}_{jg}$ in $z_{jgi}=\left|x_{jgi}-{\tilde{x}}_{jg}\right|$ is the median of the g-th subgroup. Then we performed statistical tests on each null hypothesis $H_{0i}$ , respectively. The *p*-value of each test is recorded as $L_{i}$ . We obtained a new statistic Q by combining three individual *p*-values $L_{i}$ of the statistics for each null hypothesis $H_{0i}$ .

$$Q=-2\overset{3}{\underset{i=1}{\sum }}\log L_{i}$$

Q follows the $\chi^{2}$ distribution. If $L_{i}$ is independent, $Q∼\chi^{2}\left(6\right)$ . The degree of freedom of Q is not equal to 6 in most scenarios because of the correlation of $L_{i}$ . To solve this problem, we obtained the freedom which is close to the real data distribution by $\underset{df}{\sup}\;L\left(df|Q\right)$ (more details inSupplementary Material S1).

## Data Availability

The datasets presented in this study can be found in online repositories. The names of the repository/repositories and accession number(s) can be found in the article/Supplementary Material.
