# Supplementary material for: A high-efficiency differential expression method for cancer heterogeneity using large-scale single-cell RNA-sequencing data
Source: Front Genet. 2022 Nov 29;13:1063130. doi: 10.3389/fgene.2022.1063130 (PMC9746874; doi:10.3389/fgene.2022.1063130)
Supplement: Supplementary file 1 [file DataSheet1.PDF]

Supplementary1_A high-efficiency differential expression method for cancer heterogeneity using large-scale single-cell RNA-sequencing data

Xin Yuan[1, 2] , Shuangge Ma[2 , 3] , Botao Fa[4] , Ting Wei[1 2] , Yanran Ma[1 2] , Yifan Wang[1] , Wenwen Lv[8] , Yue Zhang[1 2] , Junke Zheng[5] , Guoqiang Chen[6] , Jing Sun[7] , and Zhangsheng Yu[1 2 8 9]

[1] Department of Bioinformatics and Biostatistics, School of Life Sciences and Biotechnology, Shanghai Jiao Tong University, Shanghai, China

[2] SJTU-Yale Joint Center for Biostatistics and Data Science Organization, Shanghai Jiao Tong University, Shanghai, China

[3] Department of Biostatistics, Yale University, New Haven, United States of America

[4] Department of Biochemistry and Molecular Biology, School of Basic Medical Sciences, Xi'an Jiaotong University, Xi'an, Shanxi, China.

[5] Key Laboratory of Cell Differentiation and Apoptosis of Chinese Ministry of Education, Faculty of Basic Medicine, Shanghai Jiao Tong University School of Medicine, Shanghai, China

[6] State Key Laboratory of Oncogene and Related Gene, Shanghai Jiao Tong University School of Medicine, Shanghai, China

[7] Department of General Surgery, Shanghai Minimally Invasive Surgery Center, Ruijin Hospital, Shanghai Jiao Tong University School of Medicine, Shanghai, China

[8] Clinical Research Institute, Shanghai Jiao Tong University School of Medicine, Shanghai, China

[9] Center for Biomedical Data Science, Translational Science Institute, Shanghai Jiao Tong University School of Medicine, Shanghai, China

1. The computation of degrees of freedom of Q

According to the combination test of R. A. Fisher and K. Pearson, the new statistic $Q_i = P\left[\chi^2(df) \geqslant -2\sum_{i=1}^{3}\log L_i\right]$ follows the χ^2 distribution. If L_i is independent, the theoretical degree of freedom is 6. However, the degree of freedom of Q_i is unequal to 6 because of the correlation of L_i. We obtained the freedom by solving $\sup_{df} L(df|\boldsymbol{Q})$. $L(df|\boldsymbol{Q})$ is the likelihood function of Q_i and df. We set the $f(x)$ is the first derivatives of the logarithm of the likelihood function $L(df|\boldsymbol{Q})$. To simplify the calculation, we use the bisection method to calculate the degree of freedom parameter df that makes $f(x)$ close to zero and satisfies the precision. It is also the df that maximizes the likelihood function $L(df|\boldsymbol{Q})$ to meet the precision.

Given the interval $[a,b]$, precision: $eps = 10^{-8}$.

Initial conditions: $a < b$, and $f(a)*f(b) < 0$.

$While\ (|b-a| > eps)\ \{$

$p = \frac{a+b}{2}$

$if\ (\ f(a)*f(p) < 0)$

$b = p$

$else$

$a = p$

$\}$

output $df = \frac{a+b}{2}$

2. Simulation parameters

2.1 Parameters in Simulation 1 (Splatter)

We use the popular artificial protocol Splatter to generate various simulation datasets. The core of the Splatter is the gamma-Poisson hierarchical model. The mean expression level λ_{ij} of each gene *i* was generated from a Gamma distribution. $\lambda_{ij} \sim Gamma(\alpha, \beta)$. The final $cell \times gene$ matrix of gene means is used to generate a count matrix using a Poisson distribution. $Y_{ij} \sim Poi(\lambda_{ij})$

The DE gene generation process is as follows.

Based on the above $cell \times gene$ matrix generation process using Gamma-Poisson model, for the gene selected to the DE gene, a factor ψ_i is then multiplied by the median gene mean λ_{ij} to create new means for those genes.

$$\lambda_{ij} = I_i(\psi_i \ Med(\lambda_{ij})) + (1 - I_i)(\lambda_{ij})$$

The ψ_i is generated from a log-normal distribution. $\psi_i \sim \ln N(de.facLoc,\ de.facScale)$. I_i is an indicator that indicates whether a gene is a DE gene. I_i is generated from a Bernoulli distribution ($I_i \sim Ber(de.prob)$). The parameter $de.prob$ controls the probability that a gene will be selected to be a DE gene.

We set 1000 DE genes and 10000 non-DE genes in each simulation data. For both DE and non-DE genes, the two parameters ($Gamma(\alpha, \beta)$) of the Gamma distribution were identical. They were obtained by estimating a real single-cell dataset (Dendritic cells in the PBMC68K dataset) via the estimation process of the Splatter (Function: splatEstimate).

For non-DE genes, we set de.prob=0, meaning no gene will be selected as a DE gene. For DE genes, we set de.prob=1, which means all genes will be chosen to be DE genes. Furthermore, we simulated two different levels of differences (de.facLoc=0.3, 0.5). The high de.facLoc implies a large difference between groups. The de.facScale was estimated from the estimation process of the Splatter, like the parameters estimation of the Gamma distribution.

In all these simulations, we varied the number of cells (n=500, 1000, 2000, 5000, 10000, 20000). The simulation results of de.facLoc=0.3 showed in Fig. S2a,. The simulation results of de.facLoc=0.5showed in Fig. 2a and Fig. S2b.

For simulation1, we refered to the vignette by Splatter (https://bioconductor.riken.jp/packages/3.9/bioc/vignettes/splatter/inst/doc/splat_params.html#256_differential_expression_parameters). This vignette describes the Splat simulation model and the parameters it uses in more detail.

2.2 Parameters in Simulation 2

$S1$ is the low threshold value for the mean gene expression of the gene set for the swapping genes. The $s1$ of each real single-cell data is different in order to ensure enough genes to exchange.

type	s1_threshold
cd14mono	0.03
cd19b	0.03
cd34	0.1
THelper	0.18
TReg	0.035
NaiveT	0.05
Memory	0.04
cd56nk	0.03
CytoT	0.03
NaiveVyto	0.025
Den	0.06

3. Supplementary Figures

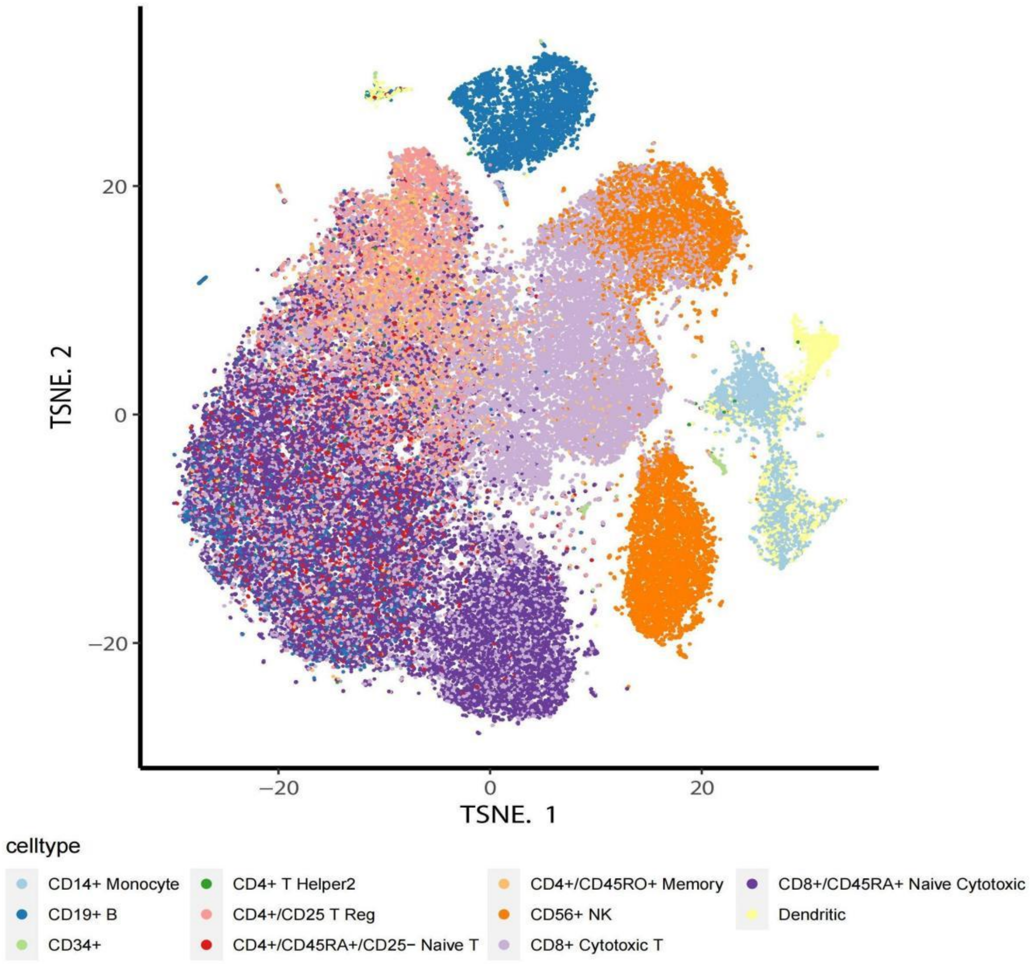

Figure S1 The tSNE plot of the PBMC68K dataset.

The PBMC68K dataset. PBMC68K is available from https://support.10xgenomics.com/single-cellgene-expression/datasets. The clustering results are obtained from the paper by Zheng et al., “Massively parallel digital transcriptional profiling of single cells”.

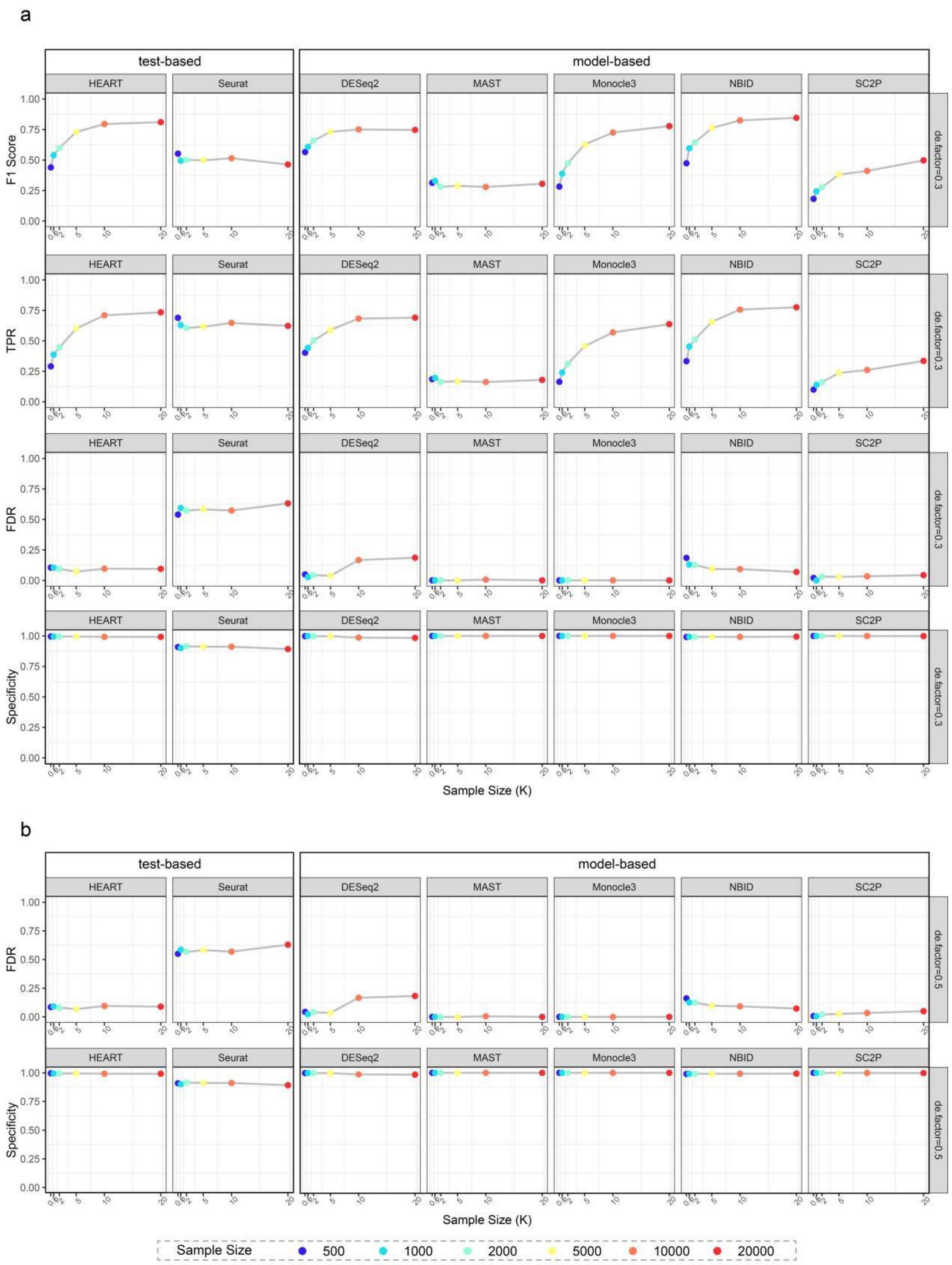

Figure S2 (**a**) F_1 scores, TPRs, FDRs, and specificities of all methods on simulation datasets in Simulation 1 (de.factor=0.3). Plots show F_1 scores (y-axis), TPRs (y-axis), FDRs (y-axis) and specificities (y-axis) for different sample sizes (x-axis) for different methods. Colorful points correspond to varied sample sizes. (**b**) FDRs and specificities of all methods on

simulation datasets in Simulation 1 (de.factor=0.5). F_1 scores and TPRs have been presented in Fig. 2a.

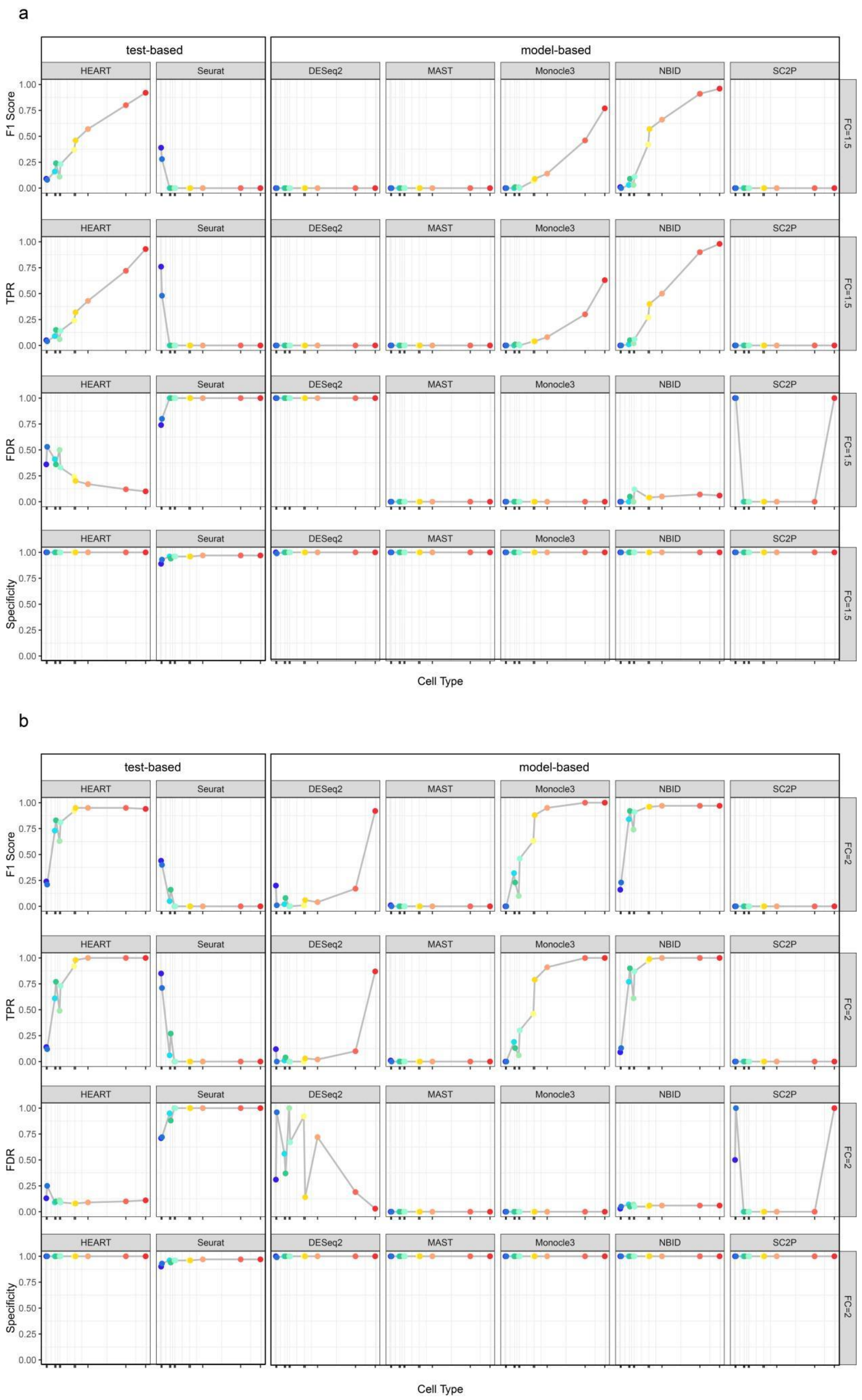
a
test-based
model-based
HEART
Seurat
DESeq2
MAST
Monocle3
NBID
SC2P
F1 Score
TPR
FDR
Specificity
FC=1.5
0.00
0.25
0.50
0.75
1.00
Cell Type
b
test-based
model-based
HEART
Seurat
DESeq2
MAST
Monocle3
NBID
SC2P
F1 Score
TPR
FDR
Specificity
FC=2
Cell Type

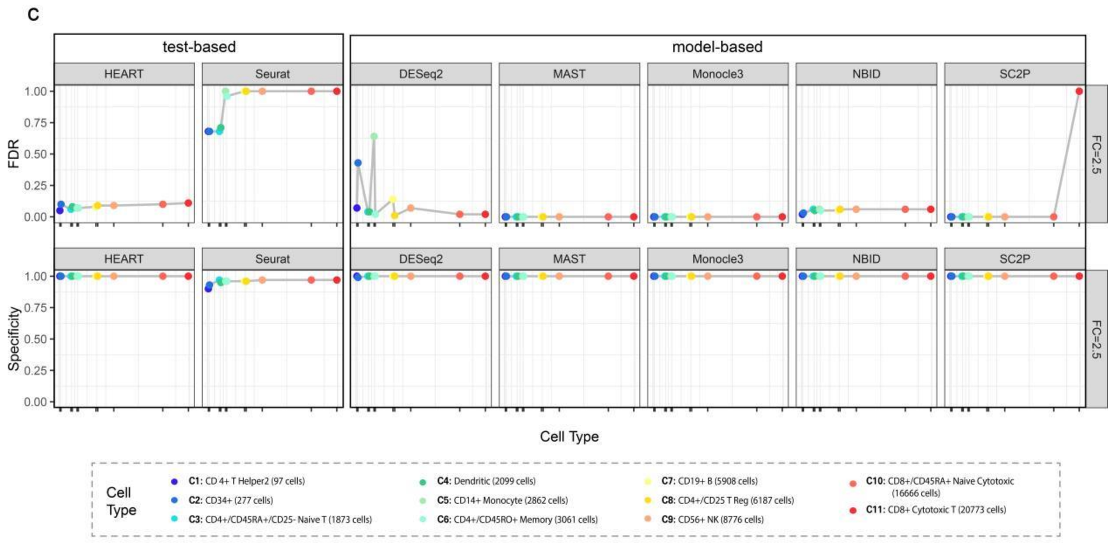

Figure S3 (**a**) F_1 scores, TPRs, FDRs, and specificities of all methods on simulation datasets in Simulation 2 (FC=1.5). Plots show F_1 scores (y-axis) TPRs (y-axis), FDRs (y-axis) and specificities (y-axis) for different source data (x-axis) for different methods. Colorful points correspond to different source datasets with different cells. (**b**) F_1 scores, TPRs, FDRs, and specificities of all methods on simulation datasets in Simulation 2 (FC=2). Plot settings are the same as in **Fig. S3a**. (**c**) FDRs and specificities of all methods on simulation datasets in Simulation 2 (FC=2.5). F_1 scores and TPRs have been presented in **Fig. 2b**.

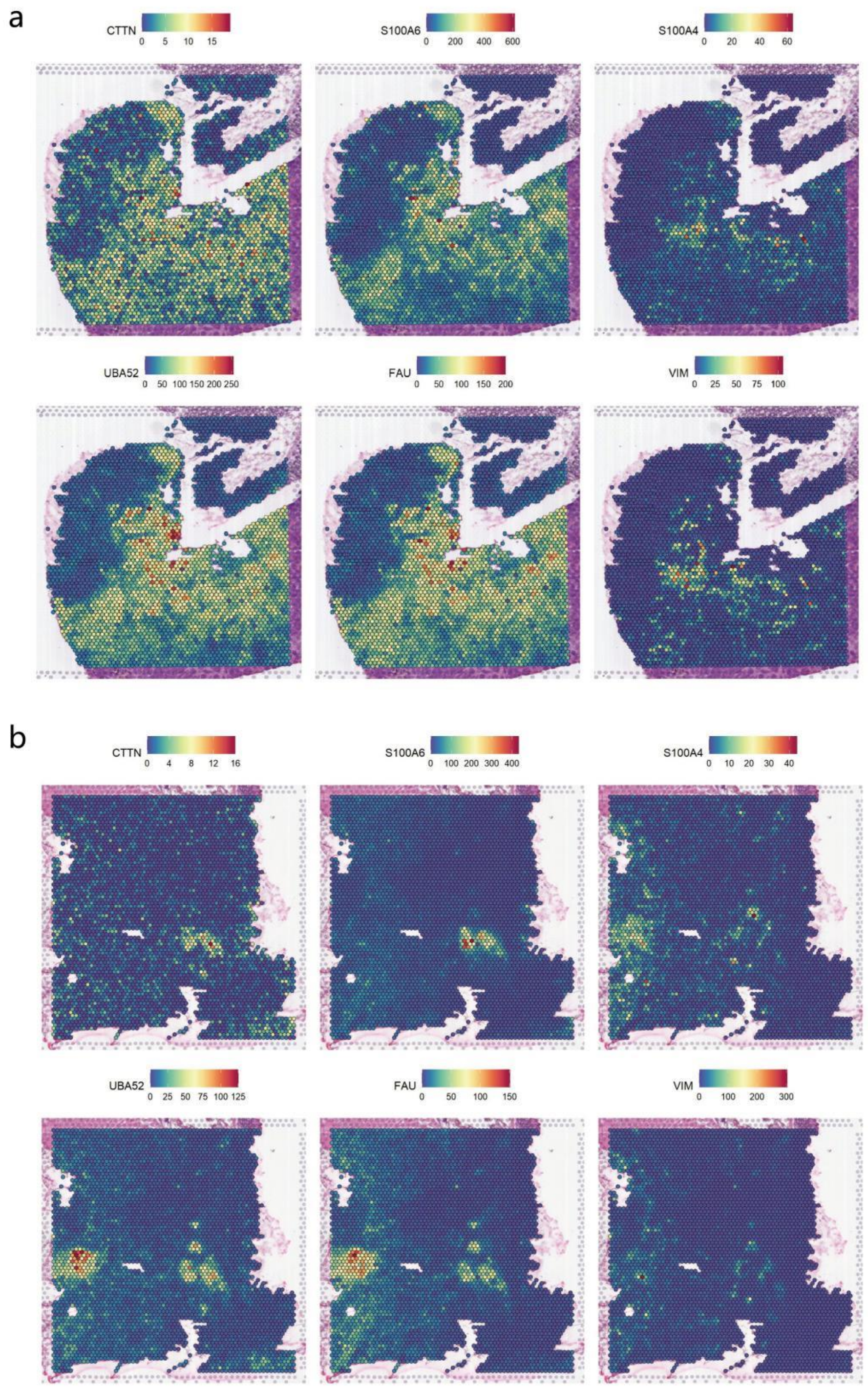

Figure S4 Potential colorectal cancer metastasis biomarkers (CTTN, S100A4, S100A6, UBA52, FAU, VIM) have similar spatial expression patterns in the spatial transcriptome data. (**a**) Spatial expression patterns of genes CTTN, S100A4, S100A6, UBA52, FAU, and VIM in the stage IV colorectal cancer patients 1. (**b**) Spatial expression patterns of genes CTTN, S100A4, S100A6, UBA52, FAU, and VIM in the stage IV colorectal cancer patients 1.

Null simulations in Simulation 2

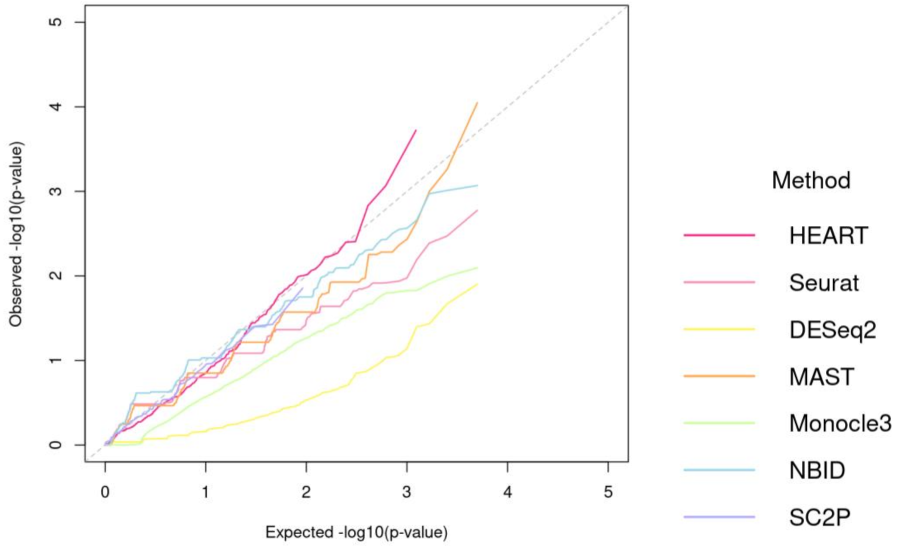

FigureS5 All methods' quantile-quantile plot of the observed -log10(p-value) against the expected -log10(p-value) under the null simulations.

We add null simulations: the real single-cell data were randomly divided into two groups without swapping gene expression. We use the quantile-quantile plot to show the results. Considering the limitations of time and computational resources, we downsample the real single-cell data (Sample size=min(1000, n_cells), n_genes=5000). Figure S4 shows the Q-Q plot of the observed -log10(p-value) against the expected -log10(p-value) under the null simulations.

New methods: SwarnSeq

We compare the computational performance of SwarnSeq with other methods in the manuscript on the simulation data (generated by Splatter: 10000 non-DE genes, 1000 DE genes with different cells). We use parallel computation to perform SwarnSeq. However, SwarnSeq is still very time-consuming, a common

problem of model-based methods. SwarnSeq takes over 22 hours to run on a dataset of 10000 cells and 11000 genes. Even SwarnSeq runs on a small dataset of 500 cells and 11000 genes, it takes 12 hours.

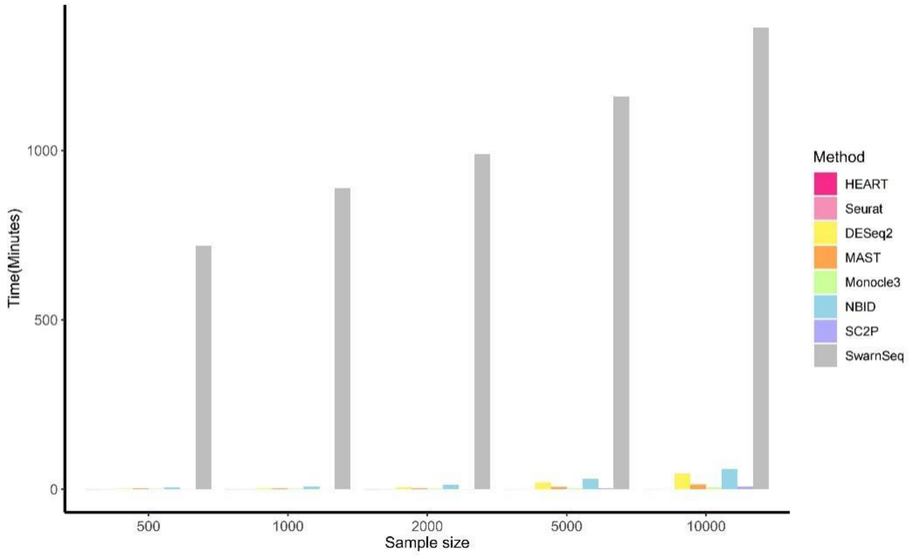

FigureS6 Computational performances of all methods on simulation data a (generated by Splatter: 10000 non-DE genes, 1000 DE genes with different cells).

In simulation 1, we simulated the small sample sizes scenarios (500, 1000, 2000 cells) when the parameter "$de.fac$" is 0.5 (Other parameters are the same as before). We compared the simulation results with the previous results (Figure3). SwarnSeq, like Seurat, tends to identify more genes as DE genes . It has high TPR but poor FDR control ability(high FDR and low specificity). Therefore, SwarnSeq has low F1 score.

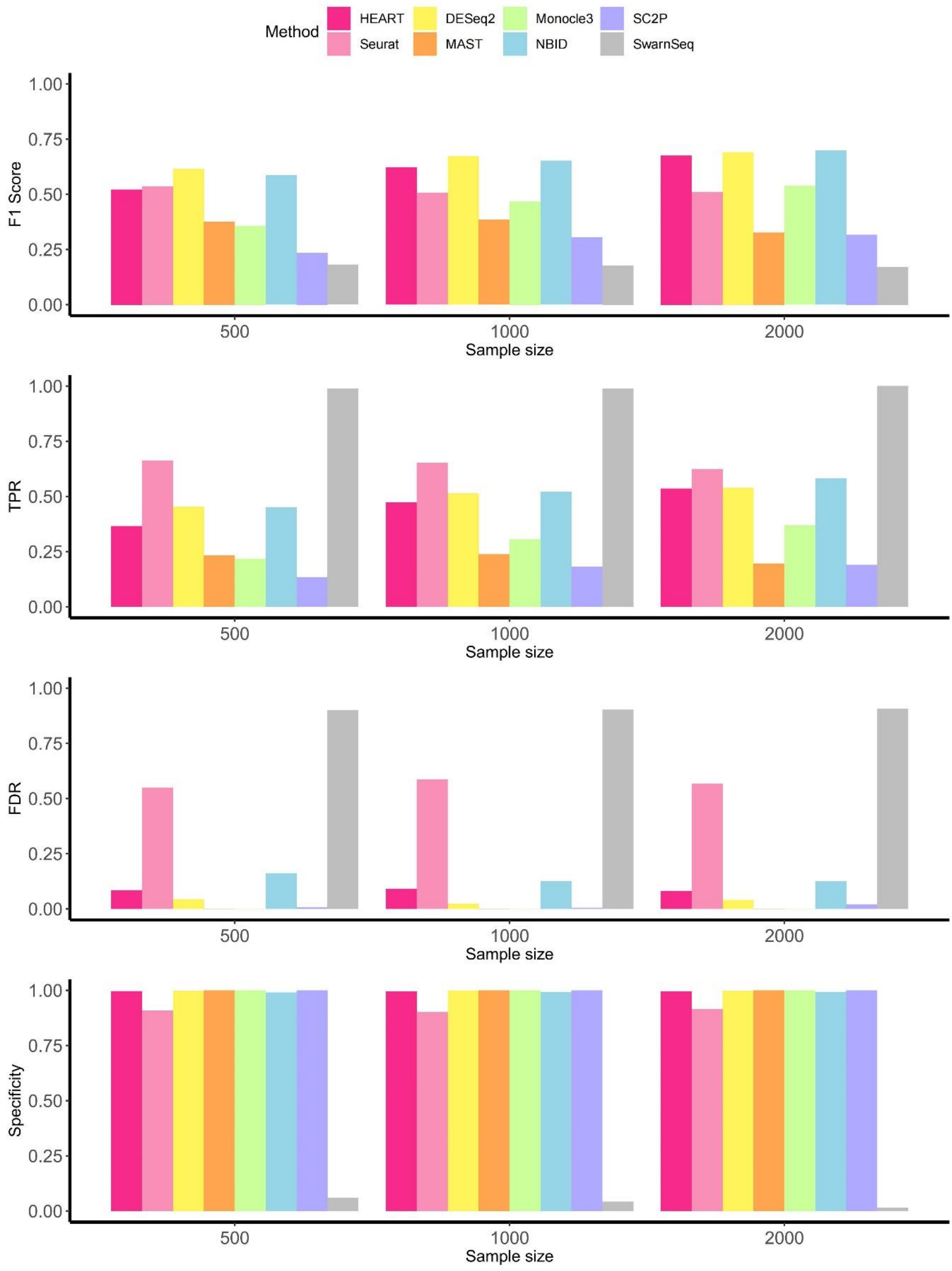

Figure S7 F_1 scores, TPRs, FDRs, and specificities of all methods on simulation datasets in Simulation 1 (de.factor=0.5).

In simulation2, we selected four real datasets for the semi-simulation. We downsampled to 1000cells for two datasets with sample sizes greater than 1000 cells. Other simulation settings were the same as in the manuscript. We compared the simulation results with the previous results (Figure4). SwarnSeq cannot distinguish between true differences and data perturbations. Similar to the results of simulation 1, SwarnSeq always identifies many genes as DE genes,so it has low F1 score, c and high TPR.

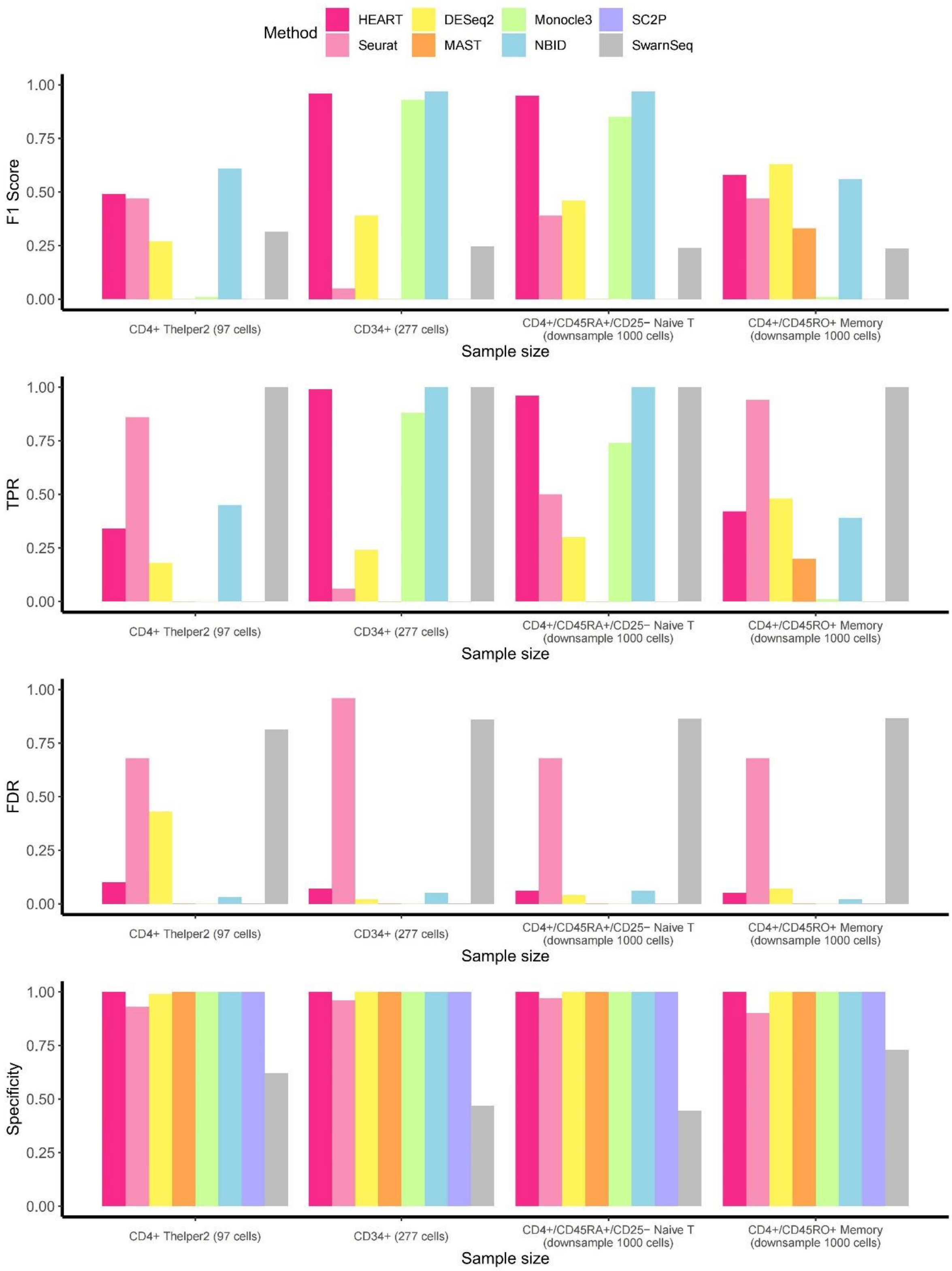

Figure S8 F_1 scores, TPRs, FDRs, and specificities of all methods on simulation datasets in Simulation 2.

Overall, SwarnSeq and NBID have similar model assumptions, but the performance of SwarnSeq is not as good as NBID.

[1]. Das S, Rai S N. SwarnSeq: An improved statistical approach for differential expression analysis of single-cell RNA-seq data[J]. Genomics, 2021, 113(3): 1308-1324.

4. Known DE genes from the literature

4.1 41 DE genes between astrocytes and oligodendrocytes.

Gene name
ABCA8
AGT
AGXT2L1
AQP4
ATP13A4
BMPR1B
C11orf9
CARNS1
CLDN11
CNDP1
ENPP2
ERMN
FGFR3
GJA1
GJB6
GPR98
HHIP
KLK6
MAG
OPALIN
RANBP3L
RNASE1
SDC4
SFXN5
SLC25A18
SLC4A4
SLCO1C1
TMEM144
UGT8

TF
DBNDD2
GPR37L1
MOBP
ACSBG1
SLC39A12
PRODH
MGST1
GFAP
FOLH1
LPAR1
CAPN3

4.2 37 DE genes between Naïve T cells and memory T cells

**Gene name**
LGALS1
S100A4
CD63
PTGER2
HLA-DRB1
GZMA
CCR6
CXCR3
IL10RA
HLA-DPA1
CD58
MAF
CD74
TNF
FAS
DUSP5
IFNG
TNFRSF1B
RGS1
GZMK
CDKN1A
SLAMF1
LGALS3
DUSP4
CFH
TBX21

IL2RB
CCL5
CXCR5
MAP3K5
HLA-DRA
CCR5
NOD2
CLECL1
LY96
STAM
TOX

4.3 HEART identifies metastatic colorectal cancer biomarkers

DE genes between two megakaryocytes, tumor and normal epitheliums, tumor and normal fibroblasts.

epitheliums	epitheliums	MKs
AAMP	A2M	EEF1A1
AATF	AAMDC	TMSB10
ABCE1	ABCA10	FAU
ABHD12	ABCA6	S100A4
ABHD17C	ABCA8	CD52
ABHD2	ABI3BP	CYBA
AC007906.2	AC020916.1	TXNIP
AC008397.1	ACTA2	RACK1
AC015712.2	ACTB	BTG1
AC103591.3	ACTG1	HLA-A
AC244090.1	ACTG2	NACA
AC245595.1	ACTN1	EEF1D
ACADVL	ACTN4	UBA52
ACOT9	ACTR2	PFDN5
ACP1	ACTR3	S100A6
ACSL3	ADAMDEC1	NKG7
ACSS1	ADAMTS12	HLA-C
ACTA2	ADAMTS4	PFN1
ACTB	ADAP2	PSME1
ACTG1	ADH1B	UQCRB
ACTL10	ADI1	HNRNPA1

ACTN4	ADIRF	COX7C
ACTR10	ADM	BTF3
ACTR3	ADRM1	PPIA
ADAM10	AEBP1	GNLY
ADAM9	AGAP2-AS1	TOMM7
ADGRG1	AHNAK	COMMD6
ADGRG6	AIDA	CORO1A
ADH5	AKR1A1	HCST
ADIPOR1	AKR1B1	IFITM2
ADNP	ALKBH7	COX4I1
ADORA2B	ANAPC11	TPT1
ADRM1	ANAPC13	IL32
AGA	ANAPC16	EEF1B2
AGPAT2	ANGPTL1	ATP5MC2
AGR2	ANGPTL2	CD48
AGR3	ANP32B	S100A10
AGTRAP	ANTXR1	GZMA
AHSA1	ANXA11	SLC25A6
AIMP1	ANXA2	ARPC3
AKR1A1	ANXA5	NOP53
AKR1B10	ANXA6	TMA7
AKR1C3	AP1S1	HLA-DPB1
AKR7A2	AP2B1	EIF3E
AKR7A3	AP2M1	SRP14
AL117339.5	AP2S1	CALM1
AL161431.1	AP3S1	CD74
AL355338.1	APEX1	EEF2
ALAS1	APOC1	CD3E
ALDH1A1	APOE	EDF1
ALDH1B1	APP	ATP5MG
ALDH1L1	APRT	CHCHD2
ALDH2	AQP8	EMP3
ALDH3B1	ARF1	MYL12B
ALDOB	ARF3	ZFP36L2
ALG1	ARF4	CST7
ALG3	ARHGAP1	HINT1
ALG5	ARHGAP29	DDX5
ANAPC13	ARHGDIA	S100A11
ANAPC16	ARHGDIB	H3F3B
ANKH	ARID5B	PNRC1
ANKRD39	ARL2	LITAF
ANO1	ARL2BP	VIM
ANP32A	ARL6IP4	LGALS1
ANP32B	ARL6IP5	COX6C

ANXA1	ARPC1A	TRAC
ANXA11	ARPC1B	GSTP1
ANXA2	ARPC2	IFITM1
ANXA3	ARPC3	PRF1
ANXA4	ARPC4	CMC1
ANXA5	ARPC5	EIF4A2
ANXA7	ARPC5L	SSR2
AOAH	ARPP19	CIRBP
AP002387.2	ASAH1	COX7A2
AP003068.2	ASF1A	CRIP1
AP003774.4	ASNA1	GMFG
AP1M2	ASPN	RAC2
AP1S1	ATF3	GZMB
AP2B1	ATF4	FGFBP2
AP2S1	ATG101	CTSW
AP3B1	ATL3	TRBC2
AP3S1	ATOX1	OST4
AP5Z1	ATP1A1	CD37
APEX1	ATP1B3	RARRES3
APIP	ATP2A2	NPM1
APMAP	ATP2B4	GZMH
APOC1	ATP5F1A	TSC22D1
APOL1	ATP5F1B	TNNC2
APOL6	ATP5F1C	ANXA1
APOPT1	ATP5F1D	CTTN
APRT	ATP5F1E	NDUFB2
AQP8	ATP5IF1	CD3D
ARCN1	ATP5MC1	MAP3K7CL
ARF1	ATP5MC2	EIF3K
ARF4	ATP5MC3	HLA-E
ARF5	ATP5MD	ID2
ARFGAP3	ATP5ME	CCNI
ARFGEF2	ATP5MF	APRT
ARHGAP29	ATP5MG	IL2RG
ARHGDIA	ATP5MPL	NDUFB7
ARID3A	ATP5PB	PPP1CA
ARID5B	ATP5PD	PTPRC
ARL1	ATP5PF	LSP1
ARL2	ATP5PO	PLAC8
ARL5A	ATP6V0B	SH3BGRL2
ARL6IP1	ATP6V0E1	CD53
ARL6IP4	ATP6V1D	CD7
ARL6IP5	ATP6V1E1	EIF3G
ARMCX6	ATP6V1F	KLRD1

ARPC1A	ATP6V1G1	PSMA7
ARPC1B	ATRAID	HNRNPA2B1
ARPC2	AUP1	CLU
ARPC3	AURKAIP1	ITGB2
ARPC4	AXL	UBC
ARPC5	B2M	LAPTM5
ARPC5L	B3GNT2	CD63
ARRDC1	BAD	IGKC
ASCL2	BAG1	ELOB
ASL	BANF1	TRBC1
ASNA1	BAX	HNRNPDL
ASPH	BCAM	RNF11
ASRGL1	BCL7B	HSPA8
ASS1	BEX3	SRSF5
ATG101	BGN	SUMO2
ATG3	BHLHE40	CLIC1
ATIC	BLOC1S1	PABPC1
ATP2A2	BMP1	TMEM40
ATP5F1A	BMP4	SPARC
ATP5F1B	BNIP3L	SNRPD2
ATP5F1C	BRK1	LIMD2
ATP5F1E	BSG	EIF3H
ATP5MC2	BTF3	ATP5F1E
ATP5MF	BTF3L4	PSMB9
ATP5PO	BTG2	TMEM59
ATP6V0B	BUD31	SARAF
ATP6V1E1	C11orf58	CFL1
ATP6V1F	C11orf96	NCOA4
ATP6V1G1	C12orf57	CA2
ATP9A	C12orf75	EIF3F
ATRAID	C15orf48	AES
AUP1	C16orf89	ARPC2
AURKAIP1	C18orf32	EVL
AXIN2	C19orf24	ATP5PO
B3GAT3	C19orf33	CNBP
B3GNT3	C19orf53	ISG20
B9D2	C19orf70	LDHB
BACE2	C1orf122	N4BP2L2
BAG1	C1orf21	POLR2L
BAG2	C1orf43	TRIR
BAG3	C1orf54	UBXN1
BAIAP2L2	C1QTNF1	RSU1
BANF1	C1QTNF6	PVALB
BAZ1A	C1R	PSAP

BBX	C1S	PTCRA
BCAP29	C2	HLA-B
BCAP31	C20orf27	VAMP8
BCL11A	C3	PGRMC1
BCL2L1	C4orf3	ERP29
BCL2L14	C4orf48	UBB
BET1	C5orf24	UBE2D3
BFAR	C6orf48	SUB1
BHLHE40	C8orf59	TSPO
BID	C9orf16	HLA-F
BLVRB	C9orf3	ACAP1
BLZF1	CA1	C19orf53
BMP4	CA4	EIF3L
BNIP1	CABIN1	PPIB
BOLA1	CADM1	CDC14B
BRCC3	CALD1	PRR13
BRD4	CALM1	SNRPG
BRK1	CALM2	TMEM258
BRMS1	CALM3	COX6B1
BROX	CALR	SLC25A3
BSPRY	CALU	SEC61B
BTF3	CAMK2G	EIF1
BTNL3	CAMLG	GSTK1
BUD23	CAMTA1	PRDX6
BUD31	CAP1	ITM2B
BZW1	CAPN2	CXCR4
C11orf24	CAPNS1	CCL4
C11orf58	CAPZA2	SEC61G
C11orf71	CAPZB	SEPT7
C12orf10	CARD16	CUTA
C12orf75	CARHSP1	HSP90AA1
C19orf24	CARMN	RBM3
C19orf33	CAST	COX5B
C19orf53	CAV1	SAP18
C1orf123	CAV2	GZMM
C1orf43	CAVIN1	GUK1
C1QBP	CAVIN3	HLA-DPA1
C2	CBR1	RBM39
C20orf27	CBX3	CCL5
C4BPB	CBX6	TAGLN2
C4orf3	CCDC102B	CTSA
C4orf48	CCDC107	NDUFA13
C6orf47	CCDC3	YBX1
C6orf48	CCDC34	SPCS1

C8orf59	CCDC59	ATP5MF
C9orf16	CCDC68	F13A1
C9orf3	CCDC85B	CD2
CA1	CCDC90B	AP001189.1
CA7	CCL11	RGS18
CAB39L	CCL13	SKP1
CACUL1	CCL2	MYH9
CACYBP	CCL5	RSL24D1
CADPS	CCL8	TRAF3IP3
CALM1	CCND1	
CALM2	CCNI	
CALM3	CCPG1	
CALR	CCT8	
CANX	CD151	
CAP1	CD24	
CAPG	CD248	
CAPN1	CD276	
CAPNS1	CD302	
CAPS	CD55	
CAPZA1	CD59	
CAPZA2	CD63	
CAPZB	CD7	
CASP1	CD9	
CASP6	CD99	
CASP8	CDC37	
CAST	CDC42	
CAVIN3	CDC42EP1	
CCDC107	CDH11	
CCDC167	CDH6	
CCDC25	CDIPT	
CCDC43	CDK2	
CCDC85B	CDK2AP2	
CCDC86	CDK4	
CCDC88B	CDKN1A	
CCL5	CEBPB	
CCNB1	CEBPD	
CCNB2	CEP85L	
CCNC	CERCAM	
CCNI	CFAP97	
CCNQ	CFD	
CCRL2	CFH	
CCT2	CFL1	
CCT3	CHCHD2	
CCT4	CHCHD5	

CCT5	CHD4
CCT6A	CHL1
CCT7	CHMP2A
CCT8	CHMP4B
CCZ1	CHMP5
CD151	CHN1
CD164	CHPF
CD177	CHPT1
CD24	CHURC1
CD320	CISD1
CD44	CISD2
CD46	CITED2
CD47	CIZ1
CD55	CKAP4
CD59	CLDN3
CD63	CLDN4
CD68	CLIC1
CD74	CLIC4
CD82	CLK1
CD9	CLMN
CD99	CLPP
CDC123	CLTA
CDC20	CLTB
CDC25B	CLTC
CDC26	CMC2
CDC37	CMTM3
CDC42	CNIH1
CDC42EP5	CNIH4
CDH3	CNN1
CDIPT	CNN2
CDK2AP2	CNN3
CDK8	CNPY2
CDKN2A	COA1
CDKN2B-AS1	COA4
CDX2	COL11A1
CEACAM6	COL12A1
CENPF	COL13A1
CENPW	COL14A1
CERS2	COL18A1
CES1	COL1A1
CETN2	COL1A2
CFAP298	COL3A1
CFAP36	COL4A1
CFB	COL4A2

CFL1	COL5A1
CGREF1	COL5A2
CHCHD2	COL6A1
CHCHD5	COL6A2
CHCHD7	COL6A3
CHID1	COL8A1
CHKB	COLEC12
CHMP3	COMMD6
CHMP4A	COMMD7
CHMP4B	COMT
CHMP4C	COPE
CHORDC1	COPS3
CHPF	COPS9
CHTF8	CORO1B
CHURC1	COX14
CIB1	COX17
CINP	COX20
CIR1	COX4I1
CIZ1	COX4I2
CKMT1A	COX5A
CKMT1B	COX5B
CKS2	COX6A1
CLCA1	COX6B1
CLCN3	COX6C
CLDN1	COX7A1
CLDN12	COX7A2
CLDN3	COX7A2L
CLDN4	COX7B
CLDND1	COX7C
CLIC1	COX8A
CLIC3	CP
CLK1	CPE
CLNS1A	CRIM1
CLPTM1	CRIP1
CLTA	CRIP2
CLTB	CRIPT
CMC2	CRTAP
CMIP	CRTC3
CMPK1	CRYAB
CMTM6	CSDE1
CMTM8	CSNK2B
CNBP	CSPG4
CNIH1	CSRP1
CNIH4	CSRP2

CNN2	CST3
CNPPD1	CSTB
CNPY2	CTDNEP1
COA3	CTDSP2
COA4	CTGF
COA6	CTHRC1
COL1A1	CTNNB1
COL4A1	CTSB
COL9A2	CTSC
COMMD2	CTSK
COMMD3	CTSZ
COMMD5	CTTN
COMMD6	CUEDC2
COMT	CUL4B
COMTD1	CUTA
COPA	CXCL1
COPB1	CXCL12
COPE	CXCL14
COPS2	CXCL2
COPS3	CXCL6
COPS5	CXCR4
COPS6	CYB5R3
COPS9	CYBA
COPZ1	CYBRD1
COQ2	CYC1
COQ4	CYCS
CORO1B	CYGB
COX20	CYR61
COX7A2L	CYSTM1
CPNE1	CYTOR
CPNE3	DAD1
CPSF2	DAZAP2
CPSF6	DBI
CRACR2B	DCN
CREB3	DCTN2
CREB3L1	DDA1
CREB3L2	DDAH2
CREG1	DDT
CRELD2	DDX5
CRIP1	DECR1
CRIPT	DEF8
CRTAP	DEGS1
CSNK2A1	DEK
CSNK2B	DEPP1

CST3	DGCR6L
CSTB	DIO3OS
CTNNA1	DKK3
CTNNB1	DLGAP4
CTNNBL1	DNAJA1
CTSA	DNAJB1
CTSB	DNAJC15
CTSH	DNAJC4
CTSO	DNPH1
CTTN	DPM2
CTU2	DPP4
CUL4A	DPY30
CUTA	DPYSL3
CWC15	DR1
CXCL1	DRAP1
CXCL3	DSTN
CXXC5	DUSP23
CYB5B	DYNC1I2
CYB5R3	DYNLL1
CYBA	DYNLRB1
CYC1	DYNLT1
CYHR1	EAPP
CYP20A1	EBF1
CYP27A1	ECM1
CYP2C18	ECM2
CYTH2	EDF1
DAD1	EDIL3
DAP	EDNRA
DAP3	EEF1A1
DBI	EEF1B2
DCTN2	EEF1D
DCTN3	EEF1G
DCTN6	EEF2
DDAH2	EFEMP1
DDOST	EFEMP2
DDRGK1	EFHD1
DDX19A	EGFL6
DDX19B	EHD2
DDX27	EI24
DECR1	EID1
DEF8	EIF1
DEGS1	EIF2S2
DENND2D	EIF3F
DERL2	EIF3G

DGAT1	EIF3H
DHRS4	EIF3I
DHRSX	EIF3J
DKC1	EIF3K
DLGAP1-AS1	EIF3L
DLGAP4	EIF3M
DLGAP5	EIF4A1
DMBT1	EIF4A2
DNAJA1	EIF4E2
DNAJB1	EIF4G2
DNAJB11	EIF5
DNAJB6	EIF5A
DNAJC1	EIF5B
DNAJC10	EIF6
DNAJC15	ELF3
DNAJC3	ELN
DNAJC7	ELOB
DNASE1L1	ELOC
DNASE2	EMC10
DNPEP	EMC4
DNTTIP1	EMC6
DPCD	EMID1
DPEP1	EMILIN1
DPM1	EMP1
DPP7	EMP3
DRAP1	ENAH
DSG2	ENC1
DSTN	ENHO
DTYMK	ENO1
DUOX2	ENSA
DUSP4	ENTPD1
DYNC1I2	ENY2
DYNLL1	EPAS1
DYNLRB1	EPCAM
DYNLT1	EPS8
EAF1	ERGIC3
EBAG9	ERH
EBNA1BP2	ERLEC1
EBPL	ERP29
ECHS1	ESAM
ECM1	ESD
ECPAS	ETFA
ECT2	EVA1B
EDF1	F3

EDN1	F8A1
EEF1A1	FAAP20
EEF1AKMT4	FABP1
EEF1D	FABP4
EEF2	FABP5
EEPD1	FADS1
EFNA3	FADS3
EGFL7	FAM107A
EHBP1L1	FAM13C
EHD1	FAM162A
EI24	FAM173A
EIF1	FAM177A1
EIF1AD	FAM198B
EIF2A	FAM20C
EIF2S2	FAM210B
EIF3D	FAM3C
EIF3E	FAM96B
EIF3F	FAU
EIF3G	FBLIM1
EIF3H	FBLN1
EIF3I	FBLN2
EIF3M	FBN1
EIF4A2	FBXO32
EIF4A3	FBXW5
EIF5B	FCGBP
EIF6	IN
ELF1	P
ELF3	FILIP1
ELOB	FILIP1L
ELOC	FIS1
ELOVL1	FKBP10
EMC4	FKBP1A
EMC6	FKBP2
EMC7	FKBP5
ENC1	FKBP8
ENO1	FLNA
ENOPH1	FLOT1
ENSA	FLYWCH2
ENY2	FMOD
EPB41L2	FOS
EPB41L4A-AS1	FOSB
EPCAM	FOXO3
EPDR1	FOXP1
EPHA2	FRMD4A

EPHB3	FSTL1
ERCC1	FSTL3
ERGIC3	FTH1
ERLEC1	FTL
ERO1A	FUNDC2
ERP44	FXR1
ESD	FXYD3
ESF1	FXYD5
ESYT2	GABARAP
ETFA	GABARAPL1
ETNK1	GABARAPL2
ETS1	GADD45A
ETS2	GADD45B
ETV4	GADD45GIP1
EXOSC3	GAPDH
EXOSC4	GDI2
EXOSC7	GEM
FAAP20	GHITM
FABP1	GJC1
FAM104A	GLMP
FAM136A	GLRX
FAM13A	GLRX3
FAM173A	GLRX5
FAM208A	GLUL
FAM210B	GNAI2
FAM27C	GNAS
FAM3A	GNB2
FAM3D	GNG11
FAM45A	GNG5
FAM49B	GOLT1B
FAM84A	GORASP2
FAM96B	GPX3
FANCC	GPX4
FANCF	GPX7
FAU	GREM1
FBXO2	GRIA4
FBXO7	GRINA
FBXO8	GSTO1
FBXW5	GSTP1
FDFT1	GTF2A2
FDPS	GTF3A
FDX1	GTF3C6
FERMT1	GUCA2A
FGFR1	GUCA2B

FGFRL1	GUCY1A1
FHL2	GUCY1B1
FIBP	GUK1
FILIP1L	GYPC
FIS1	H1F0
FKBP11	H2AFJ
FKBP2	H2AFV
FKBP3	H2AFY
FKBP8	H2AFZ
FKBP9	H3F3A
FLOT1	HAAO
FNTA	HAPLN1
FOXA3	HAPLN3
FOXP4-AS1	HCFC1R1
FRG1	HDAC2
FRGCA	HDLBP
FSCN1	HEBP2
FTL	HES1
FTSJ1	HEYL
FUBP3	HHIP
FUT2	HIF1A
FUT3	HIGD1B
FUT8	HIGD2A
FXR1	HIKESHI
FXYD5	HINT1
GABARAP	HINT2
GABARAPL2	HIP1
GADD45GIP1	HLA-A
GAL	HLA-B
GALE	HLA-C
GALNT3	HLA-E
GALNT6	HMGN1
GALNT7	HMGN2
GAPDH	HMGN3
GAPLINC	HNRNPA0
GBA	HNRNPA1
GBP3	HNRNPA2B1
GCAT	HNRNPA3
GCLC	HNRNPC
GCNT3	HNRNPDL
GDE1	HNRNPK
GDI2	HOPX
GEMIN6	HOXB-AS1
GEMIN8	HOXB2

GGH	HOXB7
GHITM	HSBP1
GID8	HSD17B10
GJB1	HSF1
GLG1	HSP90AA1
GLO1	HSP90AB1
GLRX	HSP90B1
GLRX2	HSPA1A
GLRX3	HSPA1B
GMDS	HSPA5
GMPPA	HSPA8
GMPS	HSPB1
GNAS	HSPB2
GNB2	HSPB6
GNG12	HSPD1
GNG5	HSPE1
GNPTG	HSPG2
GOLGA7	HSPH1
GOLPH3	HTRA1
GORASP2	IAH1
GOSR2	ID2
GPAA1	ID3
GPATCH4	ID4
GPR160	IDH3G
GPRC5A	IDS
GPX2	IER2
GRHPR	IER3
GRINA	IER3IP1
GRPEL1	IFI27
GSDMD	IFI27L2
GSKIP	IFI6
GSS	IFITM1
GSTK1	IFITM2
GSTO1	IFITM3
GSTO2	IFT20
GSTP1	IGF1
GTF2E2	IGFBP3
GTF2H1	IGFBP4
GTF2H5	IGFBP5
GTF2I	IGFBP6
GTF2IRD2	IGFBP7
GTF3A	IGHA2
GTF3C1	IGHG1
GTF3C5	IGHG2

GTPBP4	IGHG3
GUCA2A	IGHG4
GUCA2B	IGKV4-1
GUK1	IGLC2
GULP1	IL17B
GYG1	IL32
GZF1	ILK
GZMB	IMPDH2
H1F0	INAFM1
H2AFY	INHBA
H3F3A	INSC
HADHA	ISCU
HCCS	ISG15
HCFC1R1	ISLR
HCK	ISOC2
HDAC1	ISYNA1
HDAC2	ITGA1
HDGF	ITGAE
HDLBP	ITGAV
HEBP1	ITGB1
HEBP2	ITGB1BP1
HEMK1	ITGB5
HES6	JADE1
HEXB	JAG1
HIBADH	JCHAIN
HIF1A	JOSD2
HIST3H2A	JTB
HLA-DMA	JUNB
HLA-DMB	KANK2
HLA-DRA	KCNA5
HLA-DRB1	KCNE4
HLA-E	KCNMB1
HLA-F	KDELR2
HM13	KDELR3
HMG20B	KIF5B
HMGA1	KLHL23
HMGB1	KNOP1
HMGB3	KRT10
HMGCL	KRT19
HMGN1	KRT8
HMGN3	KRTCAP2
HNMT	KTN1
HNRNPA1	KXD1
HNRNPA2B1	LAMB2

HNRNPA3	LAMTOR1
HNRNPK	LAMTOR2
HOOK2	LAMTOR4
HOOK3	LAMTOR5
HOXA10	LASP1
HOXA9	LBH
HOXD9	LDHA
HSBP1	LDHB
HSD17B10	LGALS1
HSD17B12	LGALS3BP
HSF1	LGALS4
HSP90AA1	LGI4
HSP90AB1	LHFPL6
HSP90B1	LIFR
HSPA1A	LINC01082
HSPA1B	LINC01116
HSPA5	LINC01571
HSPA8	LINC01638
HSPB1	LITAF
HSPBP1	LMCD1
HSPD1	LMNA
HSPE1	LOXL2
HSPH1	LPP
HTATIP2	LRP1
HYAL2	LRP10
HYOU1	LRRC32
ICA1	LRRFIP1
ID1	LRRFIP2
IDH2	LSM1
IDH3B	LSM10
IDH3G	LSM2
IER3IP1	LSM4
IFI27L2	LSM5
IFI35	LSM7
IFI6	LSM8
IFITM1	LSP1
IFITM3	LTBP1
IFRD2	LTBP4
IFT172	LUM
IFT20	LURAP1L
IFT27	LY6E
IFT43	LY96
IGBP1	M6PR
IGFBP5	MAF1

IGFBP7	MAGED2
IGHG1	MALAT1
IGHG2	MANF
IGHG3	MAP1B
IGHG4	MAP3K13
IGKV4-1	MAP3K20
IGLC2	MAP3K7CL
IGLV4-69	MAP4
IL18	MAPRE2
IL1RN	MARCKS
ILF2	MATN2
ILK	MCAM
IMP3	MCL1
IMP4	MCRIP1
IMPDH2	MCRS1
INPP1	MCTS1
IRS2	MDH2
ISOC2	MDK
ISY1	MED22
ISYNA1	MEF2C
ITFG1	MEG3
ITGA6	METAP2
ITGB1BP1	METTL26
ITGB4	METTL9
ITLN1	MFAP2
JAGN1	MFAP4
JAK1	MFF
JCHAIN	MFGE8
JKAMP	MGAT1
JPT1	MGP
JTB	MGST2
JUN	MGST3
JUP	MIEN1
KANSL2	MIF
KARS	MINOS1
KAT2B	MIR4435-2HG
KAT6A	MLF2
KCNC4	MMP11
KCNE3	MMP14
KCNK6	MMP2
KCNN4	MMP23B
KCTD14	MOB1A
KDELR1	MORF4L1
KDELR2	MORF4L2

KDELR3	MPC2
KDM1A	MPG
KHDC4	MPLKIP
KIAA1522	MPZL1
KIAA1549	MRFAP1
KIF12	MRGPRF
KIF5B	MRPL12
KLF7	MRPL14
KLHDC2	MRPL16
KLK1	MRPL20
KRT17	MRPL23
KRT18	MRPL32
KRT19	MRPL33
KRT7	MRPL34
KRT8	MRPL36
KRTCAP2	MRPL41
KRTCAP3	MRPL42
KXD1	MRPL47
LACTB2	MRPL51
LAD1	MRPL52
LAMP2	MRPL54
LAMTOR1	MRPL55
LAMTOR5	MRPL57
LAP3	MRPS12
LAPTM4A	MRPS15
LAPTM4B	MRPS16
LARGE2	MRPS21
LARP6	MRPS34
LCN2	MRPS36
LDHA	MRPS6
LEFTY1	MSN
LETMD1	MSRB2
LGALS3	MSRB3
LGALS3BP	MT-ATP6
LGALS4	MT-CO1
LGR5	MT-CO2
LIMS1	MT-CO3
LINC00623	MT-CYB
LINC01315	MT-ND1
LINC02086	MT-ND2
LLPH	MT-ND3
LMAN1	MT-ND4
LMAN2	MT-ND5
LPCAT1	MT1E

LPCAT3	MT1G
LPGAT1	MT1M
LPIN1	MT1X
LRP10	MT2A
LRP5	MTCH1
LRPAP1	MTCH2
LRRC59	MTDH
LRRFIP1	MTHFD2
LRRN2	MUC2
LSM2	MUSK
LSM3	MXRA5
LSM4	MXRA8
LSM8	MYDGF
LSR	MYH11
LTA4H	MYH9
LY6E	MYL12A
LY6G6F-LY6G6D	MYL12B
LYPD8	MYL6
LYRM4	MYL6B
LYZ	MYL9
MACROD1	MYLK
MAF1	MYO1B
MAGED2	MYO1C
MAGOH	MZT2A
MAGOHB	MZT2B
MAGT1	NAA10
MAL2	NAA38
MALSU1	NACA
MANBAL	NAP1L1
MANF	NAXE
MAP1LC3A	NBDY
MAP2K3	NBEAL1
MAP3K13	NCAM1
MAP3K20	NDRG1
MAP7D1	NDRG2
MAPK13	NDUFA1
MARCKSL1	NDUFA11
MAST3	NDUFA12
MCFD2	NDUFA13
MCTS1	NDUFA3
MDH2	NDUFA4
MDK	NDUFA4L2
ME1	NDUFA5
MEAF6	NDUFA6

MET	NDUFA8
METTL23	NDUFAB1
METTL26	NDUFAF3
METTL5	NDUFAF8
METTL9	NDUFB1
MFAP2	NDUFB10
MFSD10	NDUFB11
MFSD11	NDUFB2
MGAT1	NDUFB3
MGMT	NDUFB4
MGST1	NDUFB5
MGST2	NDUFB6
MGST3	NDUFB7
MID1IP1	NDUFB8
MIEN1	NDUFB9
MIF	NDUFC1
MISP	NDUFC2
MLEC	NDUFS5
MLF2	NDUFS6
MLST8	NDUFS7
MLXIPL	NDUFS8
MMADHC	NDUFV2
MME	NEAT1
MORF4L2	NEDD8
MPDU1	NET1
MPG	NFIA
MPHOSPH8	NFIC
MPI	NFKBIA
MPST	NFKBIZ
MPZL1	NHP2
MRAP2	NINJ1
MRGBP	NMD3
MRM2	NME1
MRPL12	NME4
MRPL13	NNMT
MRPL15	NOL3
MRPL16	NOP10
MRPL18	NOP53
MRPL22	NORAD
MRPL23	NOSIP
MRPL3	NOTCH3
MRPL32	NOVA1
MRPL33	NPC2
MRPL36	NPM1

MRPL37	NPY
MRPL4	NQO2
MRPL42	NR2F2
MRPL43	NR4A1
MRPL47	NREP
MRPL48	NRGN
MRPL51	NSA2
MRPL52	NTAN1
MRPL57	NUDCD2
MRPS10	NUDT1
MRPS12	NUPR1
MRPS15	NUTF2
MRPS18B	OAT
MRPS21	OAZ1
MRPS26	OAZ2
MRPS28	OCIAD1
MRPS31	OLFML2A
MRPS34	OPTN
MRPS5	OSR1
MRPS7	OST4
MRTO4	OSTC
MS4A8	OSTF1
MT-CYB	OTULINL
MT-ND2	OXA1L
MT-ND3	P4HB
MT1G	PABPC1
MTCH1	PAIP2
MTCH2	PAK2
MTDH	PAPPA
MTIF3	PARK7
MTUS1	PARM1
MTX1	PARVA
MUC1	PCBD1
MUC2	PCBP2
MVB12A	PCNP
MYC	PCOLCE
MYDGF	PCSK6
MYEOV	PDAP1
MYH14	PDCD5
MYLIP	PDGFA
MYOF	PDGFD
MZT2A	PDGFRB
NAA15	PDIA3
NAA20	PDK4

NAA50	PDLIM1
NACA	PDLIM2
NAE1	PDLIM3
NAGLU	PDLIM4
NAPA	PDLIM5
NAPRT	PDLIM7
NCOA7	PDPN
NCOR1	PEBP1
NDFIP1	PERP
NDRG3	PET100
NDUFA12	PFDN1
NDUFA4	PFDN2
NDUFA8	PFDN4
NDUFA9	PFDN5
NDUFAB1	PFN1
NDUFAF3	PGAM1
NDUFB11	PGF
NDUFB4	PGLS
NDUFB5	PGRMC2
NDUFC2	PHB2
NDUFS2	PHGR1
NDUFS3	PHLDA1
NDUFS4	PHLDA2
NDUFS5	PHLDA3
NDUFS8	PHPT1
NDUFV2	PICALM
NECTIN2	PIGR
NEDD8	PIGT
NEIL1	PIN1
NEK2	PITX1
NEK7	PKIG
NELFE	PKM
NEU1	PLA2G16
NFAT5	PLAC9
NFU1	PLAT
NHLRC3	PLD3
NHP2	PLEC
NIFK	PLEKHH2
NINJ2	PLN
NIPA2	PLOD2
NIT2	PLP2
NKAP	PLPP1
NKD1	PLS3
NME3	PLTP

NMI	PLXDC1
NMRAL1	PLXDC2
NOB1	PMEPA1
NOL8	PMVK
NOP10	PNRC1
NOP56	PODNL1
NOP58	POLD4
NORAD	POLR2F
NOS2	POLR2I
NOSIP	POLR2J
NOXO1	POLR2J3.1
NPC2	POLR2K
NPDC1	POLR2L
NPM1	POMP
NPNT	PON2
NPRL2	POP7
NQO1	PPA1
NQO2	PPDPF
NRBP1	PPIA
NRDC	PPIB
NSA2	PPIC
NSD3	PPP1CA
NSMCE1	PPP1R11
NT5C3B	PPP1R12A
NT5DC1	PPP1R12B
NT5DC2	PPP1R14A
NTAN1	PPP1R15A
NTHL1	PPP2CA
NTMT1	PPP4C
NUBP1	PRAF2
NUCB1	PRDX1
NUCB2	PRDX5
NUDC	PRDX6
NUDT15	PRELID1
NUDT21	PRELP
NUDT22	PRKAR1A
NUP37	PRKAR2B
NUTF2	PRKRA
OASL	PRMT2
OAT	PROCR
OAZ1	PROM1
OCIAD1	PRRX1
OCIAD2	PRSS23
ODF2	PSAP

ODF3B	PSMA1
OGFR	PSMA2
OLA1	PSMA7
OLFM4	PSMB1
ORC4	PSMB2
ORMDL2	PSMB3
OST4	PSMB5
OSTC	PSMB6
OSTF1	PSMB7
OTULINL	PSMC3
OVOL1	PSMC4
P4HA1	PSMD4
P4HB	PSMD7
PABPC1	PSMD8
PACS1	PSME1
PACSIN3	PSME2
PAFAH1B2	PSMG2
PAIP1	PTCH1
PAK1	PTEN
PAK1IP1	PTGDS
PAPOLA	PTGER4
PARD6B	PTGES3
PARK7	PTGIR
PARP9	PTK2
PCBD1	PTMA
PCBP2	PTN
PCBP4	PTP4A3
PCMT1	PTPA
PCMTD1	PTPMT1
PCSK9	PTPRD-AS1
PDAP1	PTRHD1
PDCD10	PUF60
PDCD2	PYCARD
PDCD6	PYURF
PDCD6IP	QARS
PDCL3	QSOX1
PDGFA	RAB11A
PDIA3	RAB11B
PDIA4	RAB13
PDIA6	RAB1A
PDXK	RAB2A
PDZK1IP1	RAB31
PEBP1	RAB34
PERP	RAB5C

PEX16	RAB5IF
PEX2	RAB6A
PF4	RAB7A
PFDN4	RABAC1
PFKL	RABGAP1
PFKP	RAC1
PFN1	RACK1
PGAM1	RAD23A
PGD	RALBP1
PGF	RALY
PGK1	RAN
PGLS	RANBP1
PGM2L1	RAP1A
PGM3	RAP1B
PHB	RARRES1
PHB2	RARRES2
PHF12	RASL12
PHF14	RBM3
PHLDA1	RBMX
PHLDA2	RBP1
PI3	RBX1
PIGP	RCAN2
PIGT	RCN2
PIH1D1	RCN3
PIK3IP1	RCSD1
PIK3R2	REEP5
PIN4	RER1
PIP4P2	RERG
PITPNA-AS1	RERGL
PITPNB	REV3L
PITX2	REX1BD
PKIG	REXO2
PKM	RGS16
PKP3	RGS3
PKP4	RGS5
PLA2G16	RHEB
PLA2G2A	RHOA
PLBD1	RHOC
PLCB3	RHOD
PLCB4	RILPL2
PLEC	RNASEH2C
PLEK2	RND3
PLEKHA2	RNF10
PLEKHB1	RNF181

PLIN2	RNF187
PLIN3	RNF24
PLLP	RNF7
PLOD3	RNH1
PLP2	ROCK1
PLPP2	ROMO1
PLPP5	RPL10
PLSCR1	RPL10A
PLTP	RPL11
PLVAP	RPL12
PLXNB2	RPL13
PMEPA1	RPL13A
PMPCB	RPL14
PMVK	RPL15
PNPLA4	RPL17
PNRC1	RPL18
PODXL2	RPL18A
POF1B	RPL19
POLR1D	RPL21
POLR2C	RPL22
POLR2E	RPL22L1
POLR2G	RPL23
POLR2H	RPL23A
POLR2J	RPL24
POLR2K	RPL26
POLR2L	RPL27
POM121	RPL27A
POMP	RPL28
PON2	RPL29
PPA1	RPL3
PPA2	RPL30
PPARA	RPL31
PPCS	RPL32
PPIA	RPL34
PPIB	RPL35
PPIC	RPL35A
PPP1CA	RPL36
PPP1CC	RPL36A
PPP1R11	RPL36AL
PPP1R13L	RPL37
PPP1R14A	RPL37A
PPP1R14D	RPL38
PPP1R16A	RPL39
PPP1R7	RPL4

PPP2R5C	RPL41
PPP3R1	RPL5
PPP4C	RPL6
PPP4R2	RPL7
PRDX1	RPL7A
PRDX2	RPL8
PRDX4	RPL9
PRDX5	RPLP0
PREB	RPLP1
PRELID1	RPLP2
PRELID3B	RPS10
PRKAG1	RPS11
PRKCSH	RPS12
PRKRA	RPS13
PRMT2	RPS14
PRNP	RPS15
PROM1	RPS15A
PRORY	RPS16
PRPF19	RPS17
PRPF40A	RPS18
PRPSAP1	RPS19
PRR11	RPS19BP1
PRR15	RPS2
PRR5	RPS20
PRRC2C	RPS21
PRRG1	RPS23
PRRG4	RPS24
PRSS22	RPS25
PRSS23	RPS26
PRSS8	RPS27
PSAP	RPS27A
PSENEN	RPS27L
PSMA1	RPS28
PSMA2	RPS29
PSMA3	RPS3
PSMA4	RPS3A
PSMA5	RPS4X
PSMA7	RPS5
PSMB1	RPS6
PSMB2	RPS7
PSMB3	RPS8
PSMB5	RPS9
PSMB6	RPSA
PSMB7	RRAS

PSMB8	RRBP1
PSMB9	RSU1
PSMC1	RTL8A
PSMC2	RTL8C
PSMC3	RTRAF
PSMC4	RWDD1
PSMD1	S100A10
PSMD10	S100A11
PSMD11	S100A13
PSMD13	S100A16
PSMD14	S100A4
PSMD2	S100A6
PSMD3	S1PR3
PSMD4	SAP18
PSMD6	SARAF
PSMD7	SARS
PSMD8	SAT1
PSME1	SAT2
PSME2	SBDS
PSMF1	SCAND1
PSMG1	SCARA5
PSMG2	SCCPDH
PSMG3	SCP2
PTGES3	SDC2
PTMA	SDCBP
PTP4A3	SDF4
PTRH1	SDHB
PTTG1	SEC11A
PUF60	SEC23A
PUM1	SEC31A
PXDC1	SEC61A1
PYCARD	SEC61B
PYCR1	SEC61G
PYURF	SEC62
QARS	SELENOF
QPCT	SELENOH
QPRT	SELENOK
RAB10	SELENOM
RAB11A	SELENOP
RAB13	SELENOS
RAB14	SELENOT
RAB17	SELENOW
RAB1A	SEM1
RAB20	SEMA3E

RAB22A	SEPT2
RAB25	SEPT4
RAB2A	SEPT7
RAB30	SERBP1
RAB3D	SERF2
RAB5IF	SERP1
RABAC1	SERPINE2
RABEPK	SERPINF1
RACK1	SERPING1
RAD9A	SERPINH1
RALA	SERPINI1
RALBP1	SERTAD1
RALY	SF3B2
RAMMET	SF3B5
RAN	SF3B6
RANGRF	SFRP1
RAP1B	SFRP2
RAP2B	SFRP4
RARRES1	SFTA1P
RARS	SGCA
RB1CC1	SGIP1
RBCK1	SH3BGRL
RBL2	SH3BGRL3
RBM3	SH3GLB1
RBM39	SHC1
RBM42	SIGIRR
RBMX2	SIVA1
RBP1	SKIL
RBPMS	SKP1
RCN1	SLC16A3
REEP3	SLC16A4
REEP5	SLC25A3
REEP6	SLC25A39
REG4	SLC25A4
REPIN1	SLC25A5
RER1	SLC25A6
REXO2	SLC39A1
RFXANK	SLC39A4
RHBDD1	SLC48A1
RHBDL2	SLC7A2
RHEB	SLC9A3R2
RHOA	SLIRP
RHOD	SLIT3
RHOG	SLITRK6

RHOQ	SMDT1
RIC8A	SMIM10L1
RIN1	SMIM22
RIPK2	SMIM26
RMDN1	SMIM29
RNASET2	SMIM37
RNF114	SMOC2
RNF126	SNCG
RNF128	SNF8
RNF181	SNHG25
RNF186	SNRNP70
RNF43	SNRPB
RNF5	SNRPD1
RNF7	SNRPD2
RNH1	SNRPE
RNPS1	SNRPF
RPF1	SNRPG
RPF2	SNRPN
RPL10	SNU13
RPL11	SNX3
RPL12	SOCS3
RPL13	SOD1
RPL14	SOD2
RPL18	SORBS2
RPL19	SORBS3
RPL21	SORT1
RPL22L1	SOSTDC1
RPL26L1	SOX6
RPL28	SPARC
RPL29	SPARCL1
RPL3	SPATS2L
RPL30	SPECC1
RPL31	SPG21
RPL35A	SPINK1
RPL36A	SPINK2
RPL36AL	SPINT2
RPL37	SPON2
RPL38	SPTAN1
RPL39	SPTSSA
RPL4	SQSTM1
RPL7	SRP14
RPL7A	SRP19
RPL8	SRP9
RPLP0	SRSF10

RPN1	SRSF2
RPN2	SRSF3
RPS10	SRSF4
RPS11	SRSF9
RPS12	SSBP1
RPS13	SSR2
RPS15A	SSR3
RPS17	SSR4
RPS19BP1	ST13
RPS2	STMN2
RPS20	STMP1
RPS21	STOM
RPS25	STOML2
RPS26	STRAP
RPS27L	STUB1
RPS3	STX10
RPS4X	SUB1
RPS4Y1	SUCLG1
RPS5	SULF1
RPS7	SUMO1
RPUSD1	SUMO2
RPUSD3	SUMO3
RRAGA	SUPT4H1
RRAS	SURF4
RRBP1	SVBP
RRP7A	SVIL
RRP8	SWI5
RRP9	SYF2
RRS1	SYNPO
RSF1	SYNPO2
RSL1D1	SZRD1
RSL24D1	TADA3
RSU1	TAF1D
RTCA	TAGLN
RTF2	TAGLN2
RTL8A	TAPBP
RTN3	TAX1BP3
RTN4	TBC1D1
RTRAF	TBCA
RUBCNL	TBCB
RUVBL2	TCEAL3
RWDD4	TCEAL8
S100A10	TCEAL9
S100A11	TCF21

S100A13	TCIM
S100A14	TFF3
S100A16	TFPI2
S100A4	TGFB1
S100A6	TGFB1I1
S100P	TGFBI
SAAL1	TGFBR2
SAMM50	THBS2
SAP18	THOC6
SAP30BP	THY1
SARAF	TIMM13
SARS	TIMM8B
SAT1	TIMP1
SCAI	TIMP2
SCAND1	TIMP3
SCARB1	TINAGL1
SCD	TKT
SCNM1	TLE4
SCYL1	TLN1
SDC4	TM4SF1
SDCBP	TMA7
SDF2L1	TMBIM6
SDF4	TMCO1
SDHD	TMED10
SEC11A	TMED2
SEC11C	TMED3
SEC13	TMED9
SEC24D	TMEM119
SEC31A	TMEM126A
SEC61B	TMEM147
SEC61G	TMEM14C
SEC62	TMEM160
SEC63	TMEM167A
SEL1L	TMEM176A
SELENOH	TMEM176B
SELENOT	TMEM219
SELENOW	TMEM230
SEM1	TMEM256
SEMA4B	TMEM258
SERINC3	TMEM50A
SERP1	TMEM60
SERPINA1	TMSB10
SERPINB1	TMSB4X
SERPINB5	TNC

SERPINB6	TNFRSF12A
SERPINH1	TNFSF12
SESN3	TNIP1
SEZ6L2	TNIP2
SF3B2	TNS1
SF3B5	TNS3
SF3B6	TOM1
SFT2D1	TOMM20
SGSM3	TOMM22
SH3BGRL3	TOMM7
SH3GLB1	TP53TG1
SH3YL1	TPGS1
SHARPIN	TPI1
SHISA5	TPM1
SHLD1	TPM2
SIGIRR	TPM3
SKIL	TPM4
SLC12A2	TPPP3
SLC16A3	TPST1
SLC1A7	TPT1
SLC22A18	TRAPPC1
SLC22A3	TRIR
SLC25A26	TRMT112
SLC25A3	TRPT1
SLC25A6	TSC22D1
SLC2A1	TSPAN1
SLC2A8	TSPAN2
SLC35A2	TSPAN4
SLC35B2	TSPAN8
SLC35B3	TSPO
SLC35C1	TUBA1A
SLC35C2	TUBA1B
SLC35E4	TUBB
SLC39A4	TUFM
SLC3A2	TWF2
SLC40A1	TWSG1
SLC41A3	TXN
SLC43A1	TXN2
SLC44A3	TXNDC12
SLC44A4	TXNIP
SLC46A3	TXNL1
SLC50A1	TXNL4A
SLC52A2	TYMP
SLC5A1	UBA2

SLC6A6	UBA52
SLC7A6	UBB
SLC7A8	UBE2A
SLCO4A1-AS1	UBE2B
SLPI	UBE2D1
SMAGP	UBE2D2
SMC2	UBE2D3
SMG9	UBE2E1
SMIM15	UBE2E2
SMIM19	UBE2L3
SMIM24	UBL5
SMIM26	UBXN1
SMIM31	UFC1
SMIM7	UGP2
SMOC2	UQCC2
SMS	UQCC3
SMTN	UQCR10
SMYD2	UQCR11
SMYD3	UQCRB
SNAP23	UQCRC1
SND1	UQCRC2
SNHG18	UQCRFS1
SNHG25	UQCRH
SNHG7	UQCRQ
SNHG8	UROS
SNORC	USF2
SNRNP35	UXT
SNRNP70	VAMP2
SNRPA1	VAMP5
SNRPB	VAPA
SNRPB2	VARS
SNRPC	VASP
SNRPD2	VAT1
SNRPD3	VCAN
SNRPG	VCL
SNU13	VDAC1
SNX14	VDAC2
SNX17	VGLL4
SNX2	VIM
SNX3	VKORC1
SNX5	VPS29
SOD1	VSTM2A
SOD3	VTI1B
SORD	WASHC3

SOX9	WDR1
SPAG1	WDR45
SPARC	WDR83OS
SPART	WFDC1
SPATA7	WIPI1
SPCS1	WISP1
SPCS2	WTAP
SPCS3	XBP1
SPG21	YAP1
SPIN2B	YBX1
SPINK1	YIF1A
SPINK4	YIPF3
SPINT2	YPEL3
SPIRE1	YPEL5
SPOUT1	YWHAB
SPP1	YWHAE
SPRYD7	YWHAG
SPSB3	YWHAH
SPTBN1	YWHAQ
SQLE	YWHAZ
SQOR	ZBTB20
SRBD1	ZC3H15
SRD5A3	ZCCHC9
SREK1IP1	ZCRB1
SRP14	ZFAS1
SRP19	ZFHX3
SRP9	ZFP36
SRPK2	ZFP36L1
SRSF11	ZFP36L2
SRSF8	ZG16
SSB	ZMAT3
SSBP1	ZNF428
SSNA1	ZNF511
SSR1	ZNF524
SSR2	ZNF593
SSR3	ZNF688
SSR4	ZNF706
SSSCA1	ZNHIT1
ST13	ZYX
ST6GALNAC1	
STAT1	
STAU1	
STAU2	
STIP1	

STRAP
STX10
STX5
STXBP6
SUB1
SUGT1
SULT2B1
SUMO1
SUMO2
SUPT4H1
SURF4
SVBP
SVIP
SYF2
SYNE4
SYNGR2
SYPL1
TAB2
TACO1
TACSTD2
TADA3
TAF1D
TAGLN2
TALDO1
TAP1
TARDBP
TATDN1
TAX1BP1
TBC1D16
TBC1D7
TBCA
TBCB
TBL1XR1
TBPL1
TBRG1
TBX3
TCEA3
TCF20
TCF25
TCP1
TCTN3
TDG
TECR
TEN1

TERF1
TESC
TEX264
TFF3
TFG
TFPT
TGFBI
TGFBR2
TGFBR3
TGIF1
TGOLN2
THOC3
THOC7
THYN1
TIMM10
TIMM17B
TIMM23
TIMM9
TIMP1
TKT
TM2D3
TM4SF1
TM9SF2
TM9SF3
TM9SF4
TMBIM4
TMBIM6
TMC5
TMCC1
TMCO3
TMED10
TMED2
TMED3
TMED7
TMED9
TMEM102
TMEM106B
TMEM123
TMEM125
TMEM126A
TMEM126B
TMEM139
TMEM141
TMEM147

TMEM14A
TMEM14B
TMEM159
TMEM161A
TMEM165
TMEM167A
TMEM176A
TMEM176B
TMEM179B
TMEM189
TMEM205
TMEM208
TMEM214
TMEM219
TMEM230
TMEM256
TMEM258
TMEM45B
TMEM47
TMEM54
TMEM63A
TMEM87B
TMEM9
TMEM92
TMEM9B
TMF1
TMSB10
TMSB4X
TMUB1
TNFRSF12A
TNFRSF14
TNFSF15
TNNC2
TOB2
TOE1
TOMM20
TOR1AIP2
TP53I3
TP53RK
TPBG
TPD52
TPD52L1
TPI1
TPK1

TPM1
TPM2
TPM3
TPM4
TPSB2
TPSG1
TPT1
TRA2B
TRADD
TRAM1
TRAPPC1
TRAPPC3
TRIM16
TRIM2
TRIM27
TRIM7
TRIP6
TRIR
TRMT112
TRPC4AP
TRPT1
TSC22D1
TSG101
TSN
TSPAN12
TSPAN13
TSPAN5
TSPAN8
TSPO
TSR2
TSSC4
TSTA3
TTYH3
TUBA1B
TUBA1C
TUBB
TUBB4B
TUFM
TWISTNB
TXNDC12
TXNDC17
TXNL1
TXNRD1
UBA2

UBA52
UBA6
UBB
UBE2D1
UBE2D3
UBE2F
UBE2H
UBE2I
UBE2L3
UBE2L6
UBE2N
UBR2
UBXN1
UBXN6
UCA1
UCHL3
UFC1
UFD1
UFM1
UNC13D
UNC50
UNC93B1
UQCRB
UQCRC1
UQCRC2
UQCRFS1
URAD
USH1C
UTP11
UTP25
UXT
VBP1
VCP
VDAC1
VDAC2
VDAC3
VEGFB
VKORC1
VPS25
VPS28
VPS29
VPS35
VSNL1
WASHC3

WBP4
WDR24
WDR45
WDR83OS
WNT11
XBP1
XRCC6
XRN2
YAP1
YARS2
YBX1
YIF1A
YIF1B
YIPF2
YIPF3
YIPF4
YIPF6
YKT6
YPEL3
YTHDF1
YTHDF2
YWHAB
YWHAE
YWHAG
YWHAQ
YWHAZ
ZBTB1
ZBTB38
ZBTB44
ZC3H12A
ZC3H15
ZDHHC1
ZDHHC3
ZDHHC4
ZFAND1
ZFAND6
ZFAS1
ZFP36L1
ZFYVE21
ZG16
ZNF138
ZNF148
ZNF24
ZNF462

ZNF503
ZNF593
ZNF703
ZNF706
ZNF710
ZNHIT2
ZNRD1
ZNRF3
ZYX

5 More method details

5.1 Variable definitions

5.2 Distribution details
